# Hybrid Peptide-Alkoxyamine Drugs: A Strategy for the Development of a New Family of Antiplasmodial Drugs

**DOI:** 10.3390/molecules29061397

**Published:** 2024-03-21

**Authors:** Ange W. Embo-Ibouanga, Michel Nguyen, Lucie Paloque, Mathilde Coustets, Jean-Patrick Joly, Jean-Michel Augereau, Nicolas Vanthuyne, Raphaël Bikanga, Naomie Coquin, Anne Robert, Gérard Audran, Jérôme Boissier, Philippe Mellet, Françoise Benoit-Vical, Sylvain R. A. Marque

**Affiliations:** 1 Aix-Marseille University, CNRS, UMR 7273, 13007 Marseille, France; eangewilfrid@gmail.com (A.W.E.-I.); jean-patrick.joly@univ-amu.fr (J.-P.J.); 2 LCC-CNRS, Laboratoire de Chimie de Coordination and MAAP, New Antimalarial Molecules and Pharmacological Approaches, Inserm ERL 1289, Université de Toulouse, CNRS, 31077 Toulouse, France; michel.nguyen@lcc-toulouse.fr (M.N.); lucie.paloque@lcc-toulouse.fr (L.P.); jean-michel.augereau@lcc-toulouse.fr (J.-M.A.); anne.robert@lcc-toulouse.fr (A.R.); 3 Institut de Pharmacologie et de Biologie Structurale (IPBS), Université de Toulouse, CNRS, Université Toulouse III—Paul Sabatier (UPS), 31077 Toulouse, France; 4 Aix-Marseille University, CNRS, Centrale Marseille ISM2 Marseille, 13007 Marseille, France; nicolas.vanthuyne@univ-amu.fr; 5 Université des Sciences et Techniques de Masuku, LASNSOM, BP 901 Franceville, Gabon; brbikanga@hotmail.fr; 6 IHPE, University Montpellier, CNRS, Ifremer, University Perpignan Via Domitia, 66860 Perpignan, France; boissier@univ-perp.fr; 7 Magnetic Resonance of Biological Systems, UMR 5536 CNRS-University of Bordeaux, 33076 Bordeaux, France; philippe.mellet@rmsb.u-bordeaux.fr; 8 INSERM, 33076 Bordeaux, France

**Keywords:** alkoxyamine, drug resistance, prodrug, malaria, *Plasmodium*

## Abstract

The emergence and spread of drug-resistant *Plasmodium falciparum* parasites shed a serious concern on the worldwide control of malaria, the most important tropical disease in terms of mortality and morbidity. This situation has led us to consider the use of peptide-alkoxyamine derivatives as new antiplasmodial prodrugs that could potentially be efficient in the fight against resistant malaria parasites. Indeed, the peptide tag of the prodrug has been designed to be hydrolysed by parasite digestive proteases to afford highly labile alkoxyamines drugs, which spontaneously and instantaneously homolyse into two free radicals, one of which is expected to be active against *P. falciparum*. Since the parasite enzymes should trigger the production of the active drug in the parasite’s food vacuoles, our approach is summarized as “to dig its grave with its fork”. However, despite promising sub-micromolar IC_50_ values in the classical chemosensitivity assay, more in-depth tests evidenced that the anti-parasite activity of these compounds could be due to their cytostatic activity rather than a truly anti-parasitic profile, demonstrating that the antiplasmodial activity cannot be based only on measuring antiproliferative activity. It is therefore imperative to distinguish, with appropriate tests, a genuinely parasiticidal activity from a cytostatic activity.

## 1. Introduction

In 2022, around 249 million cases of malaria worldwide (a disease caused by the protozoan parasite *Plasmodium*) and 608,000 deaths occurred, mostly children under 5 years especially in Africa, promoting this disease as a major public health concern [1]. Up to now, the main series of drugs against malaria have been based on quinolines, antifolates, and artemisinin derivatives. However, drug-resistant parasites are raising and spreading worldwide, leading to a growing number of therapeutic failures [2,3]. Parasite resistance to artemisinin derivatives has now been reported in Africa, where 95% of deaths occurs, setting this continent on the edge of a tragic situation [4,5]. Consequently, research on new drug series able to circumvent parasite resistance is an absolute priority.

With the aim to develop a series of drugs expected to be active against malaria, we recently reported the synthesis and evaluation of stable alkoxyamine derivatives, which unfortunately exhibited only moderate biological activities [6]. To improve their antimalarial efficiency and, consequently, their selectivity index, we designed prodrugs based on alkoxyamine–peptide hybrids that can be activated by *Plasmodium* itself. In fact, recognition of the peptide fragment and its hydrolysis by specific parasite proteases is expected to lead to the spontaneous and instantaneous release of a potentially lethal alkyl radical within the parasite. This concept, based on the enzymatic-triggered homolysis of alkoxyamines and aiming to poison the parasite with the product it has itself generated—*to dig its grave with its fork*—has been shown somewhat successful in vitro to design antitumoral drugs [7].

*Plasmodium* is an intraerythrocytic and hematophagous parasite. Using a series of specific parasite proteases, it digests host hemoglobin inside its food vacuole to obtain supplies of amino acids required for the synthesis of its own proteins. Heme is released as a byproduct of this metabolic process. This redox-active Fe^II/III^(protoporphyrin IX) is highly toxic for every living cell, due to its ability to induce dioxygen reduction in biological conditions and, consequently, to generate reactive oxygen species, such as toxic hydroxyl radical HO•. *Plasmodium* parasites naturally detoxify heme by dimerization, followed by polymerization of the dimer to an insoluble and redox-inactive (consequently non-toxic) polymer named hemozoin [8]. Major antimalarial drugs inhibit hemozoin formation by different mechanisms, all of them leading to accumulate redox-active free heme within the parasite, thus inducing the production of lethal HO• [9,10,11].

**Scheme 1:**
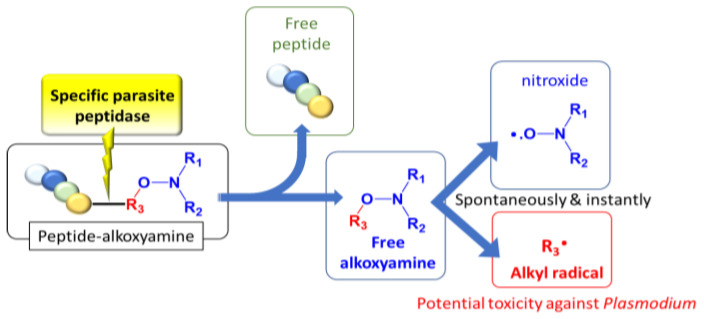
Concept: to dig its grave with its fork.

In *Plasmodium*, plasmepsins (PLMs) are a family of specific aspartic acid proteases involved in bulk protein degradation, and especially hemoglobin digestion. PLM I and II revealed an initial cleavage of hemoglobin (α_2_β_2_ tetramer) on the α chain between Phe33 and Leu34 [12]. More generally, PLMs have a rather low substrate selectivity but a strong preference for cleavage between two hydrophobic residues [12]. The design of antimalarial drugs based on the inhibition of the digestive vacuole PLM I to IV has been unsuccessful [13,14,15]. Nevertheless, PLM IX and X, located outside of the food vacuole, have also been considered as promising therapeutic targets [14], and inhibitors of plasmepsin IX exhibit a significant antimalarial efficacy even in vivo [14]. In addition, *Plasmodium* aspartic proteases are involved in various processes, such as egress from red blood cells and reinvasion. These data suggest that targeting parasite proteases may be fruitful, even if the mechanism of this efficiency remains unclear up to now. Therefore, we designed prodrugs, combining in a single molecule an alkoxyamine and a peptide selective of parasite PLMs [16]. This strategy of hybrid molecules was previously found valuable [17,18]. In the present case, the approach was based on the release of a reactive and non-selective alkyl radical resulting from the homolysis of an alkoxyamine R_1_R_2_NOR_3_, itself triggered by specific enzymatic activities of parasite plasmepsins (Scheme 1).

**Figure 1:**
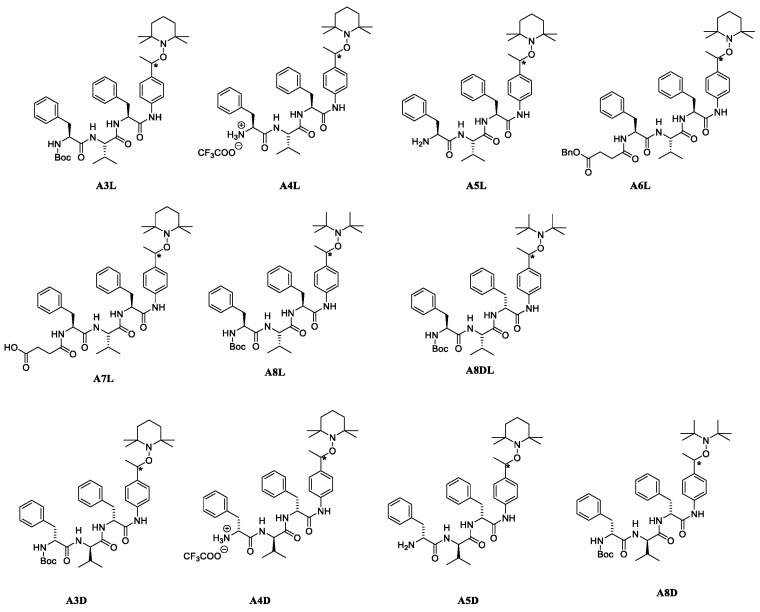
Peptide-based alkoxyamines tested as new anti-malaria prodrugs. L stands for peptides made of natural L-amino-acids and D stands for peptides made of unnatural D-amino-acids. (*) for chiral carbon on alkoxyamine moiety.

Thus, we designed prodrugs, combining in a single molecule an aryl-based alkoxyamine and the Phe-Val-Phe peptide sequence that should be a substrate of plasmepsins (Figure 1) [16]. Alkoxyamines were derived from 2,2,6,6-tetramethylpiperidine oxide (TEMPO) or di-*t*-butylnitroxide (DBNO). The role of the protecting group and of the configuration of the peptide was also considered. The IC_50_ values of these compounds on proliferative and quiescent artemisinin-resistant *Plasmodium falciparum* were evaluated, as well as their CC_50_ values on Vero cells. A preliminary docking study of the representative peptide-alkoxyamine hybrid **A3L** was carried out on the active site of the four *Plasmodium* vacuole PLMs, namely PLM I-PLM IV.

## 2. Results

### 2.1. Drug Design and Docking of Peptide-Alkoxyamine Hybrids onto PLMs I-IV

**Figure 2:**
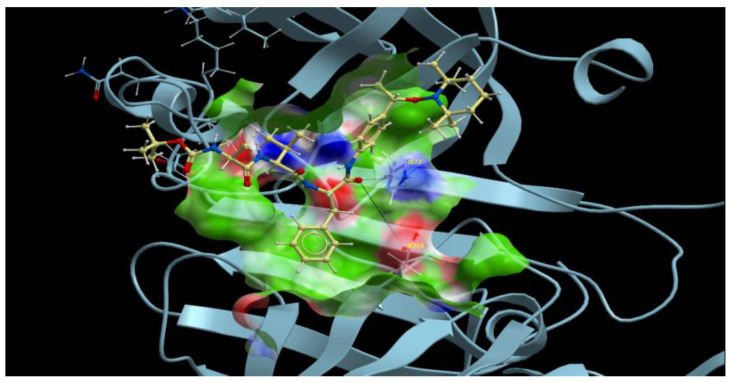
Docking of Boc-Phe-Val-Phe-anilide-TEMPO **A3L** (*S* enantiomer in yellow) on the plasmepsin III (blue, histo-aspartic proteinase PDB structure 3QVI). Dotted lines stand for the interaction with putative catalytic amino acids H32 and D215 sites.

All vacuole PLMs were able to process hemoglobin around pH 5. The initial cleavage of native hemoglobin occurred between Phe33 and Leu34 of the α chain, helping the denaturation of the highly structured hemoglobin molecule so that further proteolysis could proceed and heme could be released [12]. Thus, initially, the authors designed substrates according to the surrounding hemoglobin sequence [16]. While the P5-P1 of the best sequence for plasmepsins I and II was Lys-Glu-Phe-Val-Phe, the shorter sequence P3-P1, Phe-Val-Phe was chosen for the sake of simplicity in synthesis and for its hydrophobicity. The two configurations of alkoxyamines **A3L** (Figure 1) at the carbon adjacent of the alkoxyamine bond (star in Figure 1) were separately docked on the four vacuole PLM structures. Out of eight dockings, seven were fitted as potential substrates, and one failed, namely the *R* enantiomer with PLM II. The most convincing combinations were obtained with enantiomers *R* and *S* of **A3L** (Figure 1) on PLM III (Figure 2). P1 phenylalanine fit nicely in the protease S1 pocket, and the labile bond carbonyl pointed toward the putative catalytic amino-acids H32 and D215.

### 2.2. Preparation of the Peptide-Alkoxyamines Hybrids

**Scheme 2:**
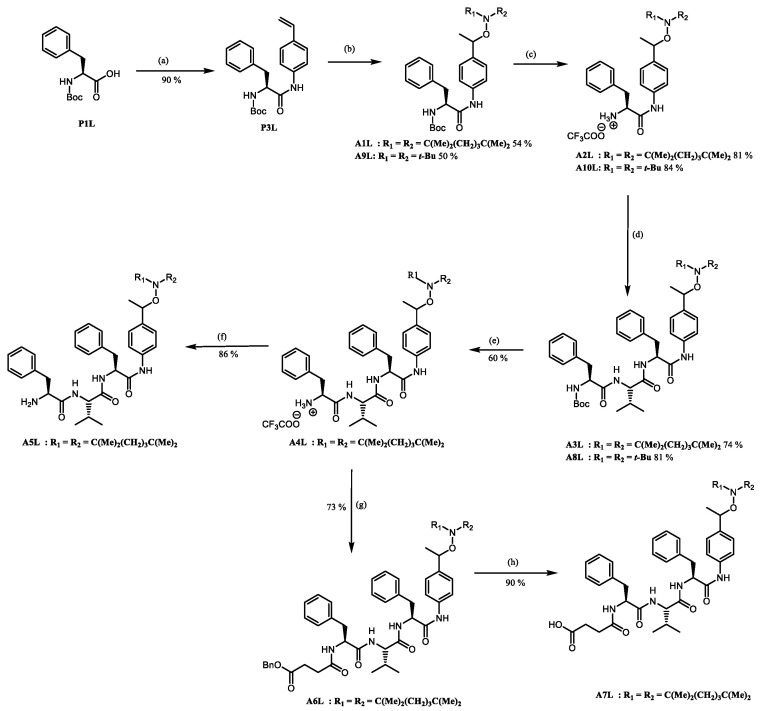
The preparation of peptide-alkoxyamine hybrids: (a) ClCOOEt, Et_3_N, 4-vinylaniline, DCM, 0 °C to −15 °C to rt, overnight; (b) salen, MnCl_2_, TEMPO, O_2_ (air), THF, rt, overnight; (c) CF_3_COOH, DCM, rt, 2 h; (d) DIPEA, HOBT, Boc-L-Phe-L-Val-OH, DCC, DCM, 0 °C to rt, overnight; (e) CF_3_COOH, DCM, rt, 4 h; (f) NaOH 1 M, DCM; (g) DIPEA, HOBT, HO-Suc-OBn, EDC.HCl, DCM, 0 °C to rt, overnight; (h) H_2_/Pd(C), DCM/MeOH (1:1, *v*:*v*), rt, 3 h.

The syntheses of hybrid molecules are displayed in Scheme 2, and details of the syntheses and characterizations are provided as Supporting Information (Schemes S1–S3) or in ref. [19].

**Scheme 3:**
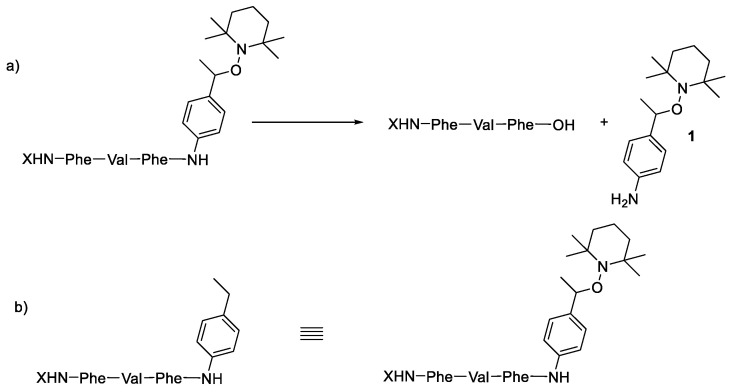
Enzymatic activity affording potentially toxic peptides (**a**) and molecules mimicking alkoxyamines (**b**).

To investigate the potential effect of the peptide moieties after the plasmepsin-mediated cleavage of the peptide-alkoxyamine linkage, two types of peptides (Scheme 3) were prepared (see SI and ref. [19]): (a) the peptides released after the hydrolysis of the hybrid-alkoxamines **P7L/D**, **P10L**, **P11L/D**, **P12L**, and **P18L** aiming to test the non-inhibiting effect of the released peptide; and (b) the peptides **P21L/D**, **P22L/D**, **P23L**, **P24L**, and **P25L** as the results of alkoxyamine homolysis without peptide hydrolysis.

The activity of these two types of peptides should be evaluated. According to Scheme 1, none of these peptides (a) or (b) are expected to be active. Any activity should consequently be due to the own cytotoxicity of the released peptide.

### 2.3. Kinetic Analysis of Alkoxyamine Bond Homolysis

**Scheme 4:**
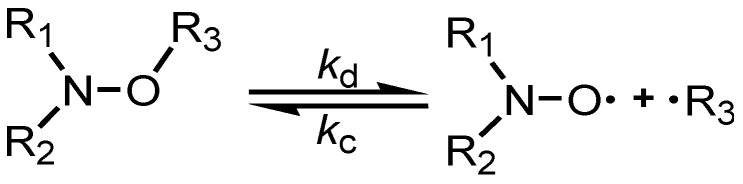
Dynamic covalent bond in alkoxyamines: *k*_d_ stands for the homolysis rate constant and *k*_c_ stands for the re-formation reaction.

The kinetic homolysis for peptide-alkoxyamines reported in Table 1 was recorded in EPR using two solvents, *tert*-butylbenzene (*t-*BuPh) or an *n-*propanol:water (1:1, *v*:*v*) mixture. The growth of nitroxide was recorded in the presence of an alkyl radical scavenger, i.e., O_2_ here, to suppress the back reaction (*k*_c_ in Scheme 4), as already reported [19].

Homolysis rate constants *k*_d_ are given by Equation (1) ([nitroxide]_∞_ = [alkoxyamine]_0_ = 0.1 mM), and the subsequent activation energy values *E*_a_ are given by Equation (2), as follows [19]:

(1) $$ln\left(\frac{{\left[nitroxide\right]}_{\infty }-{\left[nitroxide\right]}_{t}}{{\left[nitroxide\right]}_{\infty }}\right)=-k_{d}\cdot t$$

(2) $$E_{a}=-RTln\left(\frac{k_{d}}{A}\right)$$

Activation energies *E*_a_ for TEMPO-based peptide-alkoxyamine hybrids **A1L**, **A3L**–**A7L**, **A1D**, and **A3D**–**A5D** were found around 130 kJ/mol [19]. *E*_a_ values for DBNO-based-alkoxyamines **A8L/D**, **A8DL**, and **A9L/D** were close to 120 kJ/mol, as expected for aryl-DBNO-based alkoxyamines [20] and do not deserve more comments. As expected, *E*_a_ did not depend on the configuration of the peptide chain. Moreover, no difference in *E*_a_ values was observed between diastereoisomers. Thus, all alkoxyamines investigated in this article were stable under our experimental conditions.

### 2.4. Enzymatic Kinetics

**Figure 3:**
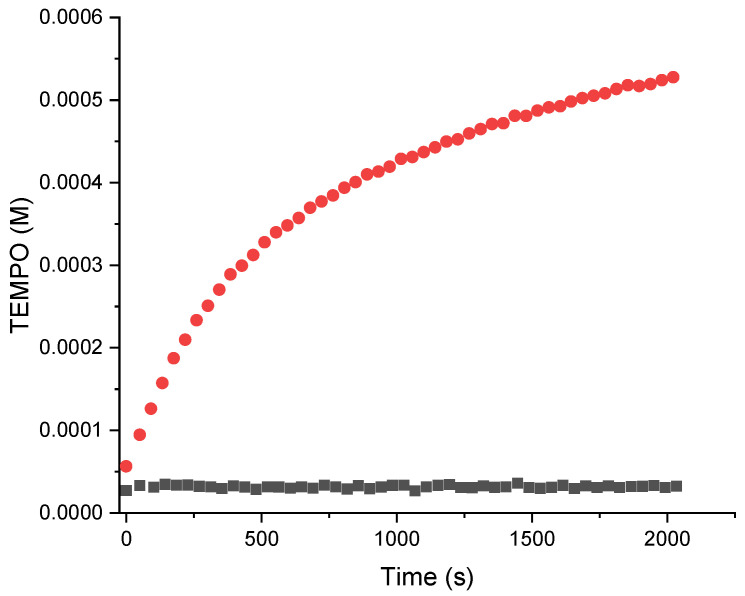
Kinetics of activation and homolysis of suc-Phe-Val-Phe-anilide-TEMPO (A7L) in the presence (red) or absence (black) of chymotrypsin.

Due to the low solubility of **A3L/D–A5L/D** in water, suitable concentrations (*ca.* 0.1 mM) for EPR detection could not be reached. Thus, **A7L** was prepared, which afforded a carboxylate function at a physiological pH. Unfortunately, no EPR signal of TEMPO could be observed in the presence of PLM I, probably because **A7L** was soluble at a pH above 5.5 and PLM I had a optimal activity around pH 5. Thus, hydrolysis of the peptide chain did not occur, and consequently, homolysis of alkoxyamine was not detectable. As other PLMs were not available, chymotrypsin, a pancreatic protease that accepts substrates with Phe in the P1 position, was used to hydrolyse **A7L**. The release of the free alkoxyamine, which decomposed spontaneously and rapidly into alkyl radical R_3_• and nitroxide R_1_R_2_NO•, and the concentration in nitroxide was monitored with EPR (Figure 3). It showed that this alkoxyamine prodrug was stable at 37 °C and activatable by chymotrypsin to indeed release free radicals. It is assumed that this result holds for all alkoxyamine derivatives reported in this article.

### 2.5. Thermal Homolysis of the Peptide-Alkoxyamine Hybrid A8L in the Presence or Absence of Hemin

**Figure 4:**
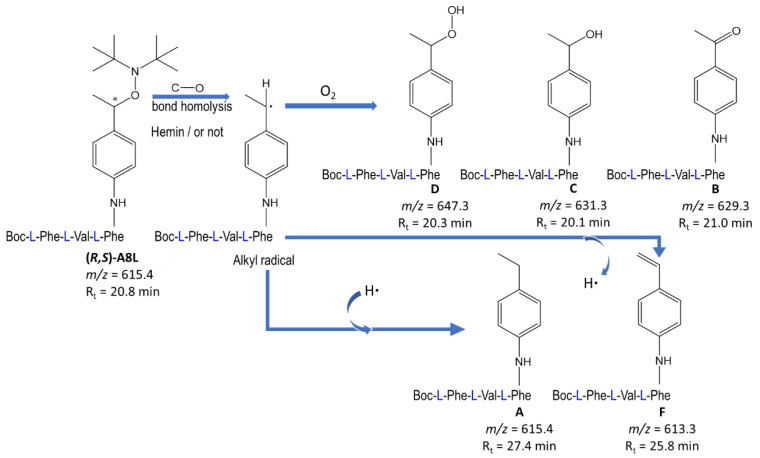
Reaction of **A8L** in DMSO at 80 °C for 1 h, in the absence of hemin [Fe(III)protoporphyrin-IX chloride].

Since a putative antimalarial mechanism of action of these hybrid drugs was the alkylation of heme within the parasite food vacuole [6], the reactivity of the hybrid having the lower IC_50_ value, namely **A8L** (Table 2), was evaluated in the presence (or absence) of heme. In the absence of any protease activity able to cleave the tripeptide fragment, the alkoxyamine C–O bond was stable at room temperature; thus, thermolysis was carried out in DMSO at 80 °C for 1 h under an air atmosphere to achieve a nearly complete reaction (88–90% of conversion of **A8L**). The reaction was monitored by LC-MS analysis, and products **A**–**F** were detected and quantified (Figure 4). In these conditions, five products showed the homolytic cleavage of the C–O bond and the subsequent reaction of its derived alkyl radical. Products **B**, **C**, and **D**, resulting from the capture of dioxygen by the alkyl radical, were the major ones, with yields of 30%, 13%, and 21%, respectively, with respect to the starting amount of **A8L**. **B** and **C** have already been detected as thermolysis products of uncoupled alkoxyamine [6].

Minor products **A** and **F**, obtained in 4% and 5%, respectively, might have resulted from the capture or from the release of H•, respectively, by the intermediate alkyl radical. The analog of **A** was already detected from uncoupled alkoxyamines [6].

In the presence of hemin (**A8L**/hemin molar ratio = 1/1), the conversion of **A8L** was similar (88–89%) after 1 h at 80 °C, and the reaction products were the same as above, except that the hydroperoxide derivative **D** was not detected, as expected, owing to the instability of hydroperoxides in the presence of metalloporphyrins [21,22]. The yields were 37% and 6% for **B** and **C**, respectively, and those of **A** and **F** were 3% and 5%, respectively, with respect to the starting amount of **A8L**. All in all, the reactivity of **A8L** was similar in the presence or in the absence of hemin. Contrary to the reactivity of antimalarial peroxides such as artemisinin, there was no alkylation of the heme macrocycle by the C-centered radical derived from the drug [6].

### 2.6. Antimalarial Activity of Peptide-Alkoxyamine Hybrids

#### 2.6.1. Antiproliferative Activity (IC_50_) against the Artemisinin-Resistant *P. falciparum* Strain, and Selectivity with Respect to Mammalian Cells (SI)

The antimalarial activity of peptide-alkoxyamine hybrids was first evaluated in the in vitro antiproliferative test (IC_50_) using the artemisinin-resistant strain F32-ART (Table 2, Table S1). The roles of the *N*-substitution of the alkoxyamine moiety (TEMPO- or DBNO-based, for example, **A3L**/**A8L**, line 1/line 10), of the configuration (L or D of the peptide, **A3L**/**A3D**, line 1/line 2), of the *N*-protection of the peptide (**A3L**/**A4L** or **A5L**, line 1/ lines 3 and 5) were examined. According to standard rules in the malaria field, only molecules having IC_50_ values below 1 μM were considered active [23].

The efficiency of the TEMPO-based hybrids was compared to that of the corresponding unbound alkoxyamine **4** (see Table 2, line 9) and peptide fragments. Then, the activities of *N*-protected or deprotected peptide-alkoxyamine hybrids were matched to the activities of the corresponding peptides, for example **P7L** and **P21L** (lines 16 and 23) for **A3L** (line 1) and **A8L** (line 10); **P12L** and **P23L** (lines 21 and 28) for **A6L** (line 7); **P10L** and **P24L** (lines 22 and 29) for **A7L** (line 8); **P21D** and **P7D** (lines 24 and 17) for **A3D** (line 2) and **A8D** (line 12); **P22L** and **P11L** (lines 25 and 18) for **A4L** (line 3); and **P22D** and **P11D** (lines 26 and 19) for **A4D** (line 4, Table 2). All the tested peptide fragments were devoid of any antimalarial activity, with IC_50_ values higher than 5 μM (Table 2, lines 16–29), regardless of configuration or protecting groups at *N*- or *C*-terminus, meaning that the activity observed was due to the alkoxyamine moiety.

In the series based on a TEMPO moiety (lines 1–7), only **A3L** (line 1) exhibited noticeable antimalarial activity, with an IC_50_ value = 300 nM, but it was still lower than that of artemisinin (IC_50_ = 31 nM, line 15). Its cytotoxicity on mammalian cells was low (CC_50_ > 50 μM), resulting in a high selectivity index (>165). This hybrid compound bears an L-Phe-Val-Phe sequence, protected at its *N*-terminus by a highly hydrophobic Boc group. The counterpart of **A3L**, bearing an unnatural peptide with D-configuration (**A3D**, line 2), was completely inactive (IC_50_ > 50 μM), suggesting the importance of the peptide recognition by a natural protease target. The activity of **A3L** was also lost upon the deprotection of the *N*-terminus, either as a salt (**A4L**, line 3) or a free base (**A5L**, line 5). The D-analogs of **A3L** after the deprotection of the *N*-terminus (**A4D**, line 4, and **A5D**, line 6) were found to be cytotoxic, with similar values of IC_50_ and CC_50_ in the range of 2.2–2.6 μM. Replacement of the Boc-protecting group by an *N*-(benzyl)succinyl **A6L** or succinyl group **A7L** resulted in a significant loss of antimalarial activity compared to **A3L** (IC_50_ in the range of 7–5 μM for **A6L**, line 7, and **A7L**, line 8, respectively).

The trend was similar in the DBNO-based series (Table 2, lines 10–14). Conjugation of the alkoxyamine with the Boc-Phe-Val-Phe peptide (**A8L**, line 10) resulted in an IC_50_ value of 270 nM, roughly the same as that of **A3L**, its analog of the TEMPO series. Similarly, a D-peptide fragment resulted in a complete loss of antimalarial activity (**A8D**, line 12, IC_50_ = 50 μM), supporting the requirement of enzymatic cleavage. This result was confirmed by the lower activity of **A8DL**, designed with the epimeric sequence Boc-L-Phe-L-Val-D-Phe, with respect to that of **A8L** (**A8DL**, line 11, IC_50_ = 0.87 μM). These results of the D-series highlighted the mandatory enzymatic hydrolysis of the peptide-tag to observe the activity of peptide-alkoxyamine hybrids. The hybrid compounds with a deprotected D-peptide fragment, AW230 and AW231, lines 13 and 14, respectively, exhibited high cytotoxicity and, consequently, low selectivity (SI ≤ 2.4).

**Table 2:** Antimalarial activity (IC_50_) and cytotoxicity against mammalian cells (CC_50_) of peptide-alkoxyamine hybrid molecules and peptide fragments.

	Prodrugs	Structure Relationship ^e^ of TEMPO-Based Alkoxyamines 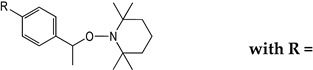	Antiplasmodial Activity ^a^ IC_50_ (µM)	Cytotoxicity ^b^ CC_50_ (µM)	Selectivity Index ^c^ CC_50_/IC_50_
1	**A3L**	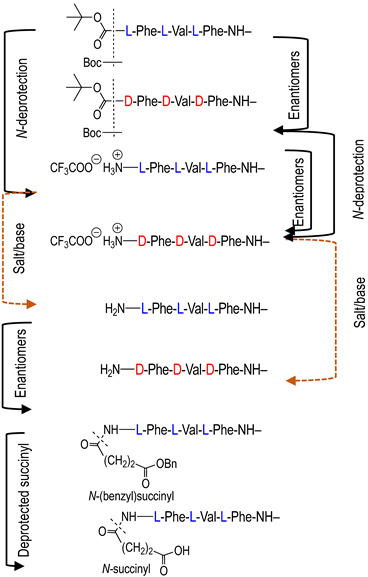	**0.30 ± 0.04**	**>50 ^i^**	**>165**
2	**A3D**		>50	>50	- ^d^
3	**A4L**		>50	>50 ^i^	- ^d^
4	**A4D**		2.3 ± 0.3	2.6 ± 0.3	1.1
5	**A5L**		>50	>50 ^i^	- ^d^
6	**A5D**		2.25 ± 0.17	2.20 ± 0.30	1.0
7	**A6L**		7.2	>50 ^i^	>6.9
8	**A7L**		5.0 ± 3.0	>50 ^i^	>10
9	**4 ^f^** ***For comparison***	H	>>10 ^e^	nd	- ^d^
	**Prodrugs**	**Structure Relationship ^e^ of DBNO-Based Alkoxyamines** 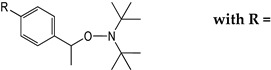	**Antiplasmodial Activity ^a^** **IC_50_ (µM)**	**Cytotoxicity ^b^** **CC_50_ (µM)**	**Selectivity Index ^c^** **CC_50_/IC_50_**
10	**A8L**	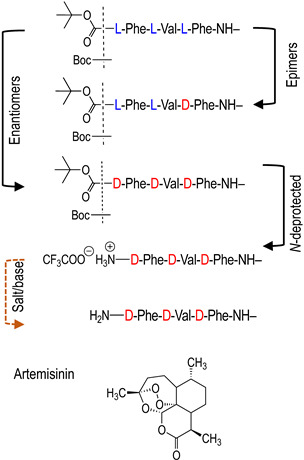	**0.27 ± 0.04**	**>50**	**>185**
11	**A8DL**		0.87 ± 0.04	>50	>57
12	**A8D**		~50	>50	- ^d^
13	**AW230**		3.73 ± 0.26	9.00 ± 1.20	2.4
14	**AW231**		4.48 ± 0.29	6.60 ± 0.40	1.5
15	**ART ^g^**		0.031 ± 0.006	>50	>1500
	**Peptides and other Comparators**	**Structure Relationship**	**Antiplasmodial Activity** ^a^ **IC_50_ (µM)**	**Cytotoxicity** ^b^ **CC_50_ (µM)**	**Selectivity Index** ^c^ **CC_50_/IC_50_**
16	**P7L**	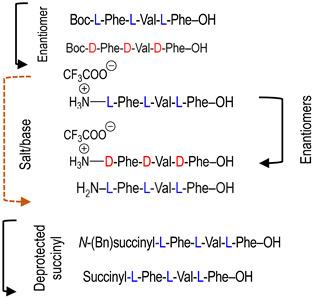	>50	>50 ^i^	- ^d^
17	**P7D**		>10	>50	- ^d^
18	**P11L**		>10	>50 ^i^	- ^d^
19	**P11D**		>10	>50	- ^d^
20	**P18L**		>5 ^h^	>25 ^h,i^	- ^d^
21	**P12L**		>10	>50 ^i^	- ^d^
22	**P10L**		>50	>50 ^i^	- ^d^
	**Peptides and Other Comparators**	**Structure Relationship**	**Antiplasmodial Activity ^a^** **IC_50_ (µM)**	**Cytotoxicity ^b^** **CC_50_ (µM)**	**Selectivity Index ^c^** **CC_50_/IC_50_**
23	**P21L**	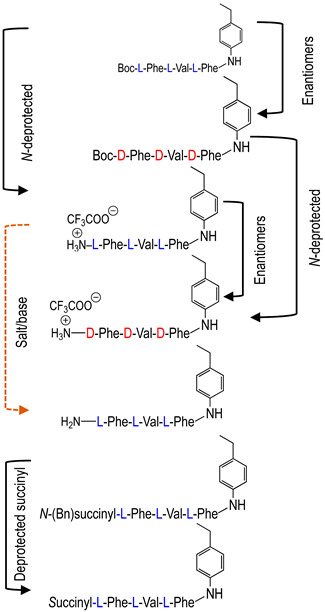	>50	>50 ^i^	- ^d^
24	**P21D**		>50	>50 ^i^	- ^d^
25	**P22L**		>10	>50 ^i^	- ^d^
26	**P22D**		10.0 ± 0.6	11.0 ± 1.6	1.1
27	**P25L**		>10	>50 ^i^	- ^d^
28	**P23L**		>10	>50 ^i^	- ^d^
29	**P24L**		>10	>50 ^i^	- ^d^

^a^ IC_50_ values corresponding to 50% inhibition of *P. falciparum* growth on the strain F32-ART [24] were determined by SYBR Green assay [25]. Values correspond to the mean ± SEM of 3 to 6 independent experiments, with technical triplicate repeats carried out for each one. ^b^ Cytotoxicity was performed on Vero cells (cell line from kidney of a normal adult African green monkey). Values correspond to the mean ± SEM of 3 to 7 independent experiments, with technical duplicate repeats carried out for each one. ^c^ Selectivity index (SI) corresponding to the cytotoxicity/antiplasmodial activity ratio. ^d^ Cannot be determined on the basis of the present data. ^e^ Enantiomer and epimer only refer to the configuration of the tripeptide Phe-Val-Phe. ^f^ Compound **9z** in ref. [6] ^g^ Antiplasmodial drug control. Artemisinin was given as a reference antimalarial drug. ^h^ Limit of solubility. ^i^ CC_50_ values reported in the submitted manuscript, ref. [19].

#### 2.6.2. Activity of Peptide-Alkoxyamine Hybrids against Artemisinin-Resistant *P. falciparum* Parasites at the Quiescent Stage

**Figure 5:**
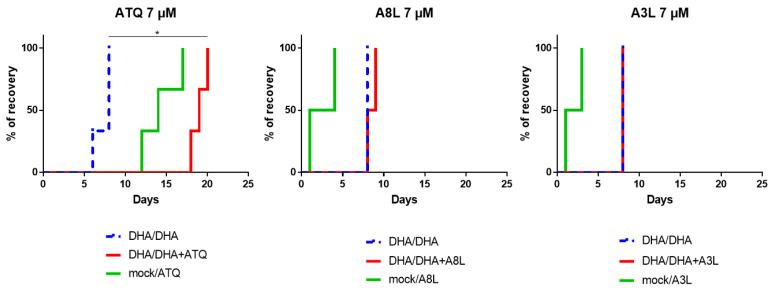
Evaluation of the activity of **A8L** and **A3L** towards F32-ART parasites in the Quiescent-stage Survival Assay (QSA) (analysis of at least 2 independent experiments). Kaplan–Meier curves show recrudescence days after different drug treatments (700 nM DHA 6 h/700 nM DHA 48 h + other compounds at 7 µM, regarding the plasma peak of atovaquone [28]). Atovaquone (ATQ) was used as positive control. Statistical significance was ascertained by using a log-rank (Mantel-Cox) test (* = *p*-value < 0.05).

Artemisinin resistance was mediated by a quiescence (dormancy) mechanism, allowing parasites to withstand drug exposure [2,24,26]. However, this quiescent state deeply modified parasite metabolism and, thus, the availability of therapeutic targets. Thus, an antiplasmodial drug can be active against a parasite in a proliferative state but can lose its activity against quiescent parasites, as it was demonstrated with chloroquine [27]. That is why we investigated the activity of the two best compounds on parasite proliferative state, **A8L** and **A3L**, against ART-resistant parasites in a quiescent state using the Quiescent-stage Survival Assay (QSA) [27] (Figure 5).

In this assay, parasites were exposed to dihydroartemisinin (DHA) alone to induce quiescence, prior the addition of the drug to be tested for 48 h, and then the parasite recrudescence was monitored over a maximum of 4 weeks. Activity against quiescent parasites was evidenced by a delay in recrudescence time over 6 days between the conditions “DHA 6 h / DHA 48 h” and “DHA 6 h/(DHA + molecule) 48 h”. Atovaquone was used as a positive control [27], and its activity against quiescent parasites was here confirmed, as expected, by an extended delay of 11 days in recrudescence time (Figure 5). By contrast, no activity of **A8L** and **A3L** against quiescent parasites was evidenced in the QSA, with similar recrudescence time between the conditions “DHA 6 h/DHA 48 h” and “DHA 6 h/(DHA + molecule) 48 h”. Surprisingly, 48 h treatments at 7 µM with each of these two drugs alone led to very short recrudescence times (1 to 4 days) when compounds were tested at concentrations 20 times higher than their IC_50_ values. To understand this phenomenon, we explored the parasite response during treatment with these hybrid compounds.

#### 2.6.3. Moment of Action of Peptide-Alkoxyamine Hybrids Regarding the Erythrocytic Parasite Cell Cycle

**Figure 6:**
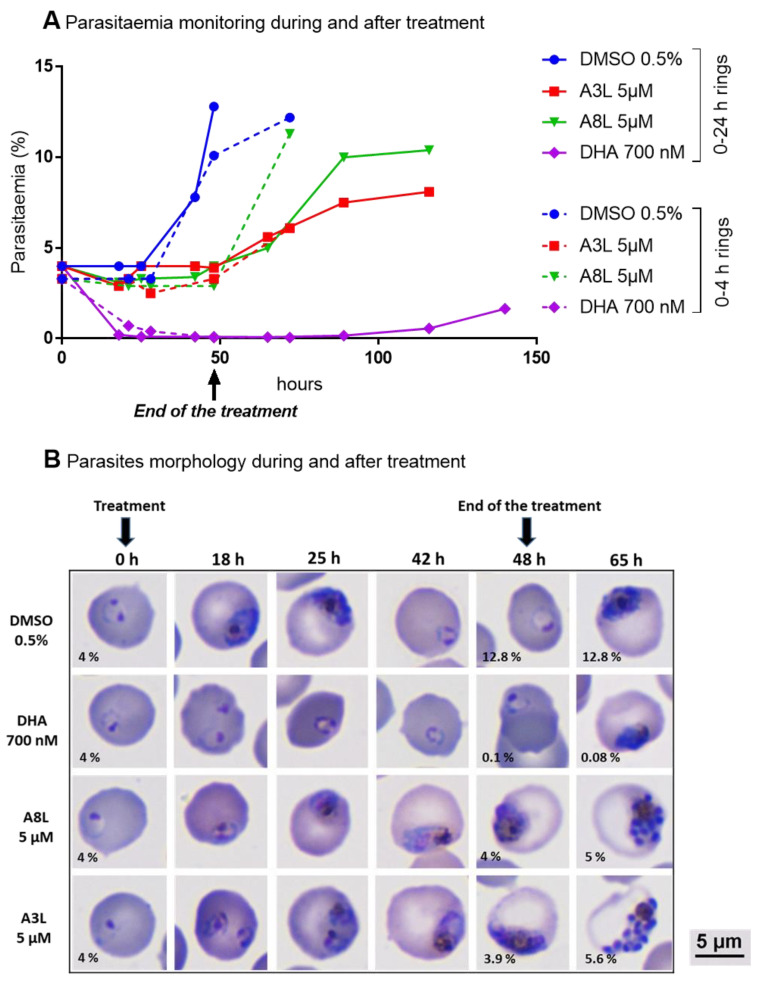
Evaluation of the in vitro efficacy of DHA, **A3L**, **A8L**, and DMSO as vehicle controls regarding the erythrocytic cell cycle of F32-ART parasites. DHA was used as an antiplasmodial drug reference. (**A**) Parasitemia monitoring on parasite smear and (**B**) the morphology of parasites during and after drug exposure of ring-stage parasites (0–24 h old at the time of the start of treatment, DiffQuick-stained blood smear, magnification 100 with immersion oil). Parasitemia after 48 h and 65 h of treatments was indicated on each corresponding picture.

Parasitemia monitoring, as well as the observation of F32-ART parasite morphology upon drug exposure, evidenced a cytostatic-like effect of the two best peptide-alkoxyamines in the chemosensitivity assay (**A3L** and **A8L**). In fact, during the 48 h of exposure to the drug, the normal timing of the parasite’s cell cycle was slowed down; the parasites were able to pass from the ring stage to the trophozoite stage, but they did not reach the next schizont stage and were unable to re-invade new red blood cells at the end of the normal duration (48 h) of the parasite’s intra-erythrocytic cycle (Figure 6).

**Figure 7:**
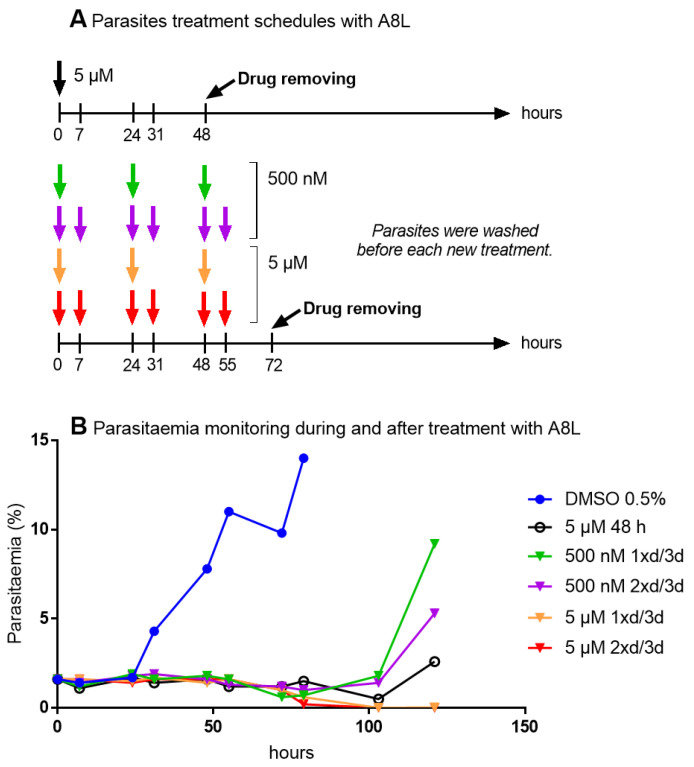
The evaluation of activity of different treatment schedules with **A8L** (1xd/3d: once a day for three days, 2xd/3d: twice a day for three days) against F32-TEM parasites. (**A**) Treatment schedules. (**B**) Parasitemia monitoring during and after the drug exposure of ring-stage parasites (0–24 h old at the start of the treatment).

This result explains the high difference in parasitemia between untreated (DMSO control) and treated parasites (whatever the hybrid molecule used) at 48 h and, therefore, the IC_50_ values obtained. By contrast, DHA, used as an antiplasmodial drug reference and known for its strong and fast action, showed a high parasitemia decrease. The parasite recrudescence observed after drug removal was due to the quiescence phenomenon of a parasite sub-population, as the parasites used were artemisinin-resistant. To confirm the cytostatic-like effect of **A3L** and **A8L** hybrid compounds, a new assay was conducted with **A8L** on the F32-TEM parasites that were artemisinin-sensitive. A similar cytostatic-like effect was observed in F32-TEM parasites subjected to the same treatment regimen, but these ART-sensitive parasites resumed a normal growth more slowly after the end of the treatment than ART-resistant parasites (Figure 7, “5 µM 48 h” condition).

**Figure 8:**
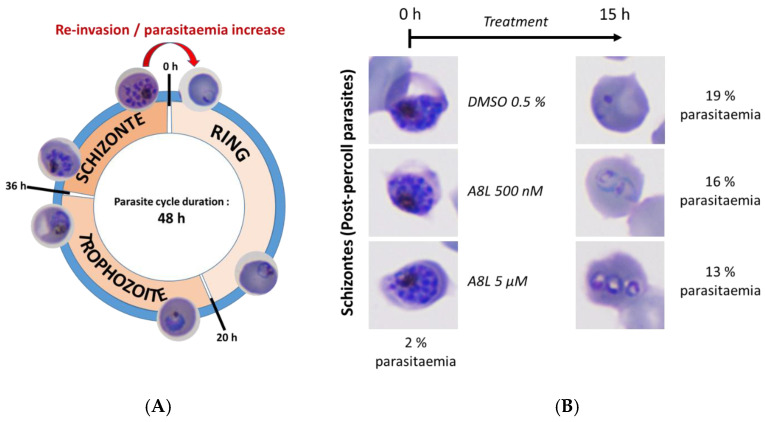
Evaluation of the in vitro efficacy of **A8L** on the re-invasion step of the parasite cycle. (**A**) Description of the different steps of the intraerythrocytic phase of *P. falciparum* life cycle. (**B**) Parasite morphology and parasitemia monitoring before and after the drug exposure of post-percoll schizonts (DiffQuick-stained blood smear, magnification 100 with immersion oil).

Repeated exposure of the parasites to **A8L** for at least three days led to the elimination of parasites in a dose-dependent manner. We wondered if this activity was due to a specific effect of the drug toward the schizont stage and re-invasion step of the parasite life cycle. However, treatment of parasites at the schizont stage for 15 h with **A8L** had no effect on parasite viability or development (Figure 8), highlighting that the mechanism of action of **A8L** has yet to be identified.

## 3. Discussion

We previously reported the synthesis and evaluation of stable alkoxyamine derivatives, that unfortunately exhibited only moderate biological activities [6]. To improve their antimalarial efficiency and, consequently, their selectivity index, we designed prodrugs containing an alkoxyamine fragment linked to a short peptide sequence. The recognition and hydrolysis of the peptide fragment by specific proteases of *Plasmodium* is expected to release an unstable alkoxyamine within the parasite, whose instantaneous homolysis should produce a lethal alkyl radical. Thus, the prodrug activity is expected to only take place within the parasite, thus leading to an improved selectivity. In fact, this mechanism should be similar to the antimalarial activity of artemisinin (and synthetic peroxides) that is triggered by Fe^II^-heme released by the parasite-induced hemoglobin digestion, leading the parasite to be poisoned by the waste it has generated itself. On these bases, hybrid compounds were synthesized and evaluated against the malaria parasite. These compounds were based on a reactive alkoxyamine covalently bound to a peptide sequence that was supposed to be the substrate of specific parasite proteases.

The *N*-Boc-protected peptide-alkoxyamine hybrids **A3L** and **A8L** exhibited significant anti-proliferative activity against *P. falciparum* with IC_50_ values of 270 and 300 nM, respectively, and a high selectivity index (SI > 165). Deprotection of the *N*-terminus of the peptide moiety, or unnatural D-peptides, led to inactive compounds. This indicates that the *N*-Boc protection and natural L-configuration of the peptide were crucial for the antimalarial activity and, probably, for the recognition and cleavage of the peptide sequence by *Plasmodium* proteases, thus validating the design of these peptide-alkoxyamine hybrids. Nevertheless, the antimalarial activities of **A3L** and **A8L** were roughly ten times lower than that of the reference drug artemisinin, whose IC_50_ value was 31 nM (Table 2, line 15). The thermolysis of **A8L** carried out in the presence of hemin produced the corresponding alkyl radical, but this radical failed to alkylate the hemin macrocycle (Figure 4). Thus, the antiproliferative activity of **A8L** cannot be assigned to the accumulation of redox-active heme derivatives, such as for artemisinin. Thus, the detailed molecular mechanism of the antimalarial activity of **A3L** and **A8L** is not determined yet.

Moreover, it is important to note that most studies evaluating the antimalarial activity of compounds are limited to IC_50_ values, and this gives fragmented and incomplete information leading to false claim efficiency. Indeed, the antiproliferative activity evidenced here with these hybrid compounds was due to a cytostatic but not lethal effect of the drugs in the time-frame of experiment. However, the repeated exposure of parasites to peptide-alkoxyamine hybrids for at least three days (**A8L** 5 µM once a day for three days) was effective to totally kill the total parasite population.

Thus, the next issue is the design of new hybrids with an enhanced antimalarial activity (lower IC_50_, with a non-cytostatic but lethal effect). This might be achieved by using a longer peptide sequence, more specific to parasite proteases. Indeed, hybrids investigated here already displayed some kinetic features requested for the efficiency of our approach (Scheme 1), namely the spontaneous and instantaneous homolysis of the alkoxyamine O–N bond that is supposed to dramatically limit the diffusion of active alkoxyamine, and the high reactivity of generated alkyl radicals with (life-times < 1 μs), to a few dozens of nanometers. However, the moderate anti-proliferative and cytostatic activity against *Plasmodium* suggests that prodrugs must comply with additional factors for an efficient antimalarial activity.

Owing to some similarities between hemoglobin digestion by *Plasmodium* and *Schistosoma* parasites [6,29,30], the hybrid peptide-alkoxyamine derivatives were also evaluated in vitro against *S. mansoni* [22]. The most active peptide-alkoxyamine derivatives against *S. mansoni* were **A4L** and **A5L** (at 100 μg/mL, 50% of a schistosomal population was killed in 2 h), while these hybrids were totally inactive against *Plasmodium*. This suggests that suitable kinetic homolysis parameters of the drugs are a necessary but not sufficient condition to anticipate their biological activity. Additional parameters dependent on each parasite should obviously be taken into consideration. More generally, *Plasmodium* being a parasite able to enter dormancy, which is, by definition, a non-proliferative stage, the “classical” anti-proliferative test providing IC_50_ values was insufficient to evaluate the actual antimalarial activity of molecules, which requires additional tests, such as, for example, recrudescence tests.

## 4. Materials and Methods

### 4.1. Docking

Molecular docking experiments were conducted using the following PDB structures: Plasmepsin I: 3qs1; Plasmepsin II: 4y6m; Plasmepsin III: 3qvi; Plasmepsin IV: 5i70. The alkoxyamine **A5L** stereoisomers *R* and *S* were built and docked separately on the four plasmepsins using ICM-pro software, version 3.9-3a (Molsoft LLC, San Diego, CA, USA).

### 4.2. Enzyme Kinetics of Alkoxyamine Activation

Kinetics were performed in HEPES 50 mM buffer containing 0.15 mM NaCl at 37 °C. Then, 2.5 mM of hemin (Santa Cruz biotechnology, Heidelberg, Germany) was added to scavenge the alkyl radicals. In one, kinetic Chymotrypsin (Worthington, OH, USA) was added. Activation and homolysis were monitored by reading the concentrations of released TEMPO using, versus time, a calibrated EPR spectrometer (EMX Nano, Bruker, Wissembourg, France).

### 4.3. Thermolysis of A8L in the Absence or in the Presence of Hemin

In a test tube equipped with a magnetic stirrer, 20 μL of a stock solution of **A8L** (10 mM in DMSO) was added to 180 μL of DMSO. Alternatively, 20 μL of the stock solution of **A8L** (10 mM in DMSO) was added, followed by 20 μL of the stock solution of hemin (10 mM in DMSO) and 160 μL of DMSO. The solutions were heated at 80 °C for 1 h. LC-MS analyses were performed at t_0_ and at 1 h as follows: a 5 μL aliquot of each reaction mixture was diluted in DMSO (95 μL), and 7 μL of the diluted solutions was injected. LC-MS analyses were carried out on a Waters Xevo G2 QTof equipped with a Waters X-Bridge C18 column (5 μm, 4.6 × 100 mm), plus an Interchim precolumn (3 μm, 4.0 × 10 mm). The eluents were: (A) H_2_O/formic acid, 100/0.1, *v*/*v*; (B) CH_3_OH/formic acid, 100/0.1, *v*/*v*. The elution gradient was from A/B = 100/0 to A/B = 20/80 in 15 min, then A/B = 20/80 was continued until 30 min. The flow rate was 0.4 mL/min. UV detection was at 260 nm. Retention times and detected *m/z* values (amu) were as follows: JP2310, 20.8 min, 758.5 (MH^+^), 702.4 (fragment); A, 27.4 min, 615.4 (MH^+^); B, 21.2 min, 629.3 (MH^+^); C, 20.1 min, 631.4 (MH^+^); D, 20.3 min, 647.3 (MH^+^); F, 25.7 min, 613.3 (MH^+^); hemin, 17.3 min, 616.2 (M^+^).

### 4.4. Parasite Culture

The F32-ART (artemisinin-resistant) strain and the F32-TEM (artemisinin-sensitive strain) [24] were cultured according to Trager and Jensen [31] in RPMI 1640 medium (Fisher Scientific, Illkirch, France) supplemented with 5% human serum (Etablissement français du Sang, Toulouse, France) at 2% hematocrit with type O human blood (Etablissement français du Sang, Toulouse, France), 37 °C, and 5% CO_2_ in a humidified atmosphere.

### 4.5. Biological Activity of New Compounds

IC_50_ values corresponding to 50% inhibition of *P. falciparum* growth were determined by SYBR Green assay, and the assessment of cytotoxicity was performed on Vero cells (cell line from the kidney of a normal adult African green monkey), as previously published [6]. The selectivity index corresponded to the cytotoxicity/activity ratio.

### 4.6. Quiescent Stage Survival Assay (QSA)

The QSA was performed according to Reyser et al. [27]. Briefly, ART-resistant ring-stage parasites at 3% parasitemia were exposed for 6 h to 700 nM of DHA (dihydroartemisinin) to induce quiescence or were exposed to no drugs (control condition). After that, the compound to be tested was added in both conditions at 48 h. At the end of the treatment, the drugs were washed off with RPMI-1640, and parasites were replaced in drug-free conditions. Blood smears were performed to follow parasitemia until the day the cultures reached their starting parasitemia, defined as the recrudescence day. If after 30 days no parasite recrudescence was observed, then the data were censored. Data analysis was performed using Kaplan–Meier survival curves. Statistical significance was ascertained by using a log-rank (Mantel-Cox) test using GraphPad Prism 7 software (San Diego, CA, USA).

### 4.7. Microscopic Examination of Parasites upon Drug Exposure

The effect of hybrid compounds against *Plasmodium* parasites was assessed by the microscopic examination of parasite morphology upon drug exposure. For this purpose, parasites were synchronized at 0–4 h age by percoll-sorbitol treatment, 0–24 h age by sorbitol treatment, or at schizont stage by percoll treatment at the start of the experiment. Different treatment schedules were tested. In the case of multiple doses, parasite cultures were washed before each new drug addition.

## 5. Conclusions

In the classical chemosensitivity assay, **A3L** and **A8L** exhibited significant activity against *P. falciparum* with IC_50_ values at 270 and 300 nM, respectively, and a high selectivity index (SI > 165). Conversely, their analogues containing the unnatural D-peptide sequence (**A3D** and **A8D**, respectively) were inactive. This indicates that the natural L-configuration of the peptide moiety was crucial for the antimalarial activity and, probably, for the recognition and cleavage of the peptide sequence by *Plasmodium* proteases. These results highlight the mandatory protease activity of the parasite to initiate the antimalarial activity (Scheme 1), thus validating the design of these peptide-alkoxyamine hybrids. They consequently validate our approach that aimed to use, and not to inhibit, suppress, or disturb, a mandatory enzymatic activity of the parasite (the fork) to promote the release of a drug, here, the alkoxyamine that homolysed into an alkyl radical exhibiting anti-proliferative activity toward the parasite, by unselective ways (to dig the parasite grave).

Moreover, the *N-*Boc protection of the amine terminus of **A3L** and **A8L** was also required, as assessed by the lack of antimalarial activity of **A4L**, the *N*-deprotected derivative of **A3L**, or **A5L** and **A6L**, bearing *N*-succinyl derivatives as protecting groups. Until now, the exact role of the *N*-Boc group has not been further investigated.

Importantly, it should be noted that, despite rather low IC_50_ values, the effect of **A3L** and **A8L** was cytostatic, but not lethal, toward *Plasmodium* in usual test conditions. Repeated exposure of the parasites to **A8L** for at least three days was required to eliminate parasites in a dose-dependent manner. More generally, *Plasmodium* being a parasite able to enter dormancy which is, by definition, a non-proliferative stage, the “classical” anti-proliferative test providing IC_50_ values is insufficient to evaluate the actual antimalarial activity of molecules, which requires additional tests, such as recrudescence tests.

The next challenge is the design of peptide-alkoxyamine hybrids with an enhanced antimalarial activity, with a non-cytostatic but lethal effect on *Plasmodium*. This might be achieved by using a peptide sequence more specific to parasitic proteases and an optimized alkoxyamine able to kill effectively the parasite.

## Figures and Tables

**Table 1:** Experimental temperatures (*T*), homolysis rate constants *k*_d_, activation energies *E*_a_ in *t-*BuPh, and *n*-PrOH/H_2_O for peptide-alkoxyamine hybrids.

Peptide-Alkoxyamine Hybrids	*T* (°C) *^a^*	*k*_d_ (10^−4^ s^−1^) *^a,b,c^*	*E*_a_ (kJ/mol) *^b,d^*	*T* (°C) *^e^*	*k*_d_ (10^−4^ s^−1^) *^b,c,e^*	*E_a_* (kJ/mol) *^b,d,e^*
A8L	97.7	22.1	120.9	75.0	1.7	120.9
A8DL	91.3	19.0	119.3	83	10	118.4
A9L	107.9	72.3	120.5	81	6.7	118.9
A8D	91.1	10	121.1	90.2	22.0	118.5
A9D	89	9.7	120.8	85.2	11.0	118.9

*^a^* In *t*-BuPh *^b^* Values measured for a mixture of diastereosisomers. *^c^* Given by Equation (1). *^d^* Given by Equation (2), and an averaged frequency factor was used *A* = 2.4 · 10^14^ s^−1^, *R* = 8.314 J·K^−1^·mol^−1^, *k*_d_, and *T* are given in columns 2, 3, 5, and 6 in Table 1. *^e^* In water/*n*-PrOH, 1/1, *v*/*v*.

## Data Availability

The data presented in this study are available in the article.

## Supplementary Materials

The following supporting information can be downloaded at https://www.mdpi.com/article/10.3390/molecules29061397/s1: Schemes S1–S3: Preparation of the peptide tags; Table S1: Antimalarial activity and cytotoxicity against mammalian cells of peptide fragments.
