# Supplementary material for: Hybrid Peptide-Alkoxyamine Drugs: A Strategy for the Development of a New Family of Antiplasmodial Drugs
Source: Molecules. 2024 Mar 21;29(6):1397. doi: 10.3390/molecules29061397 (PMC10974622; doi:10.3390/molecules29061397)
Supplement: Supplementary file 1 [file molecules-29-01397-s001.zip › molecules-2890525-supplementary.pdf]

Supporting Information

Preparation of peptide fragments

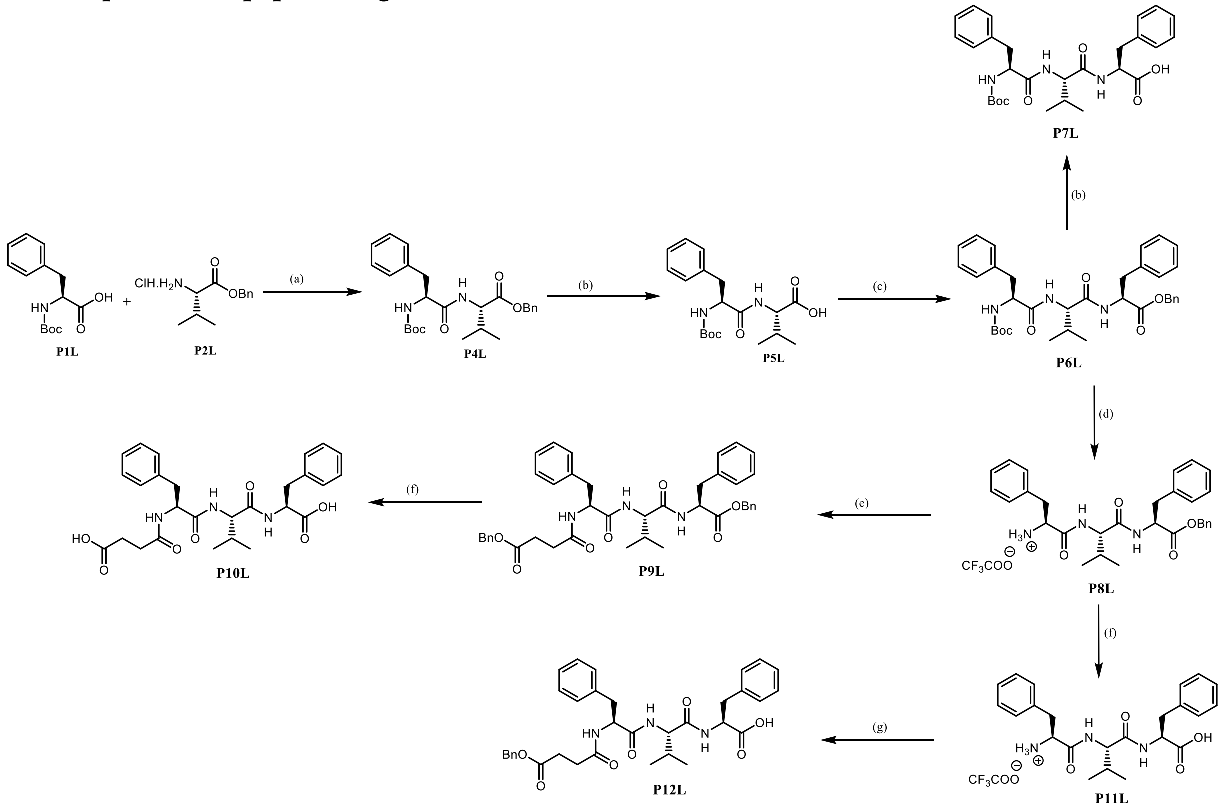

Scheme S1. Preparation of the peptide tag: (a) DIPEA, HOBT, DCC, DCM, 0 °C to rt, overnight, 97%; (b) H_2/Pd(C), DCM/MeOH (v/v 1:1), rt, 3h, 94%, (c) DIPEA, HOBT, HCl.NH_2-L-Phe-OBn, DCC, DCM, 0 °C to rt, overnight, 73%; (d) CF_3COOH, DCM, rt, 4h, 79%; (e) DIPEA, HOBT, HO-Suc-OBn, EDC.HCl, DCM, 0 °C to rt, overnight, 62%; (f) H_2/Pd(C), MeOH, rt, 3h, 94%, (g) HOBT, HO-Suc-OBn, EDC.HCl, DCM, 0 °C to rt, overnight, 47%.

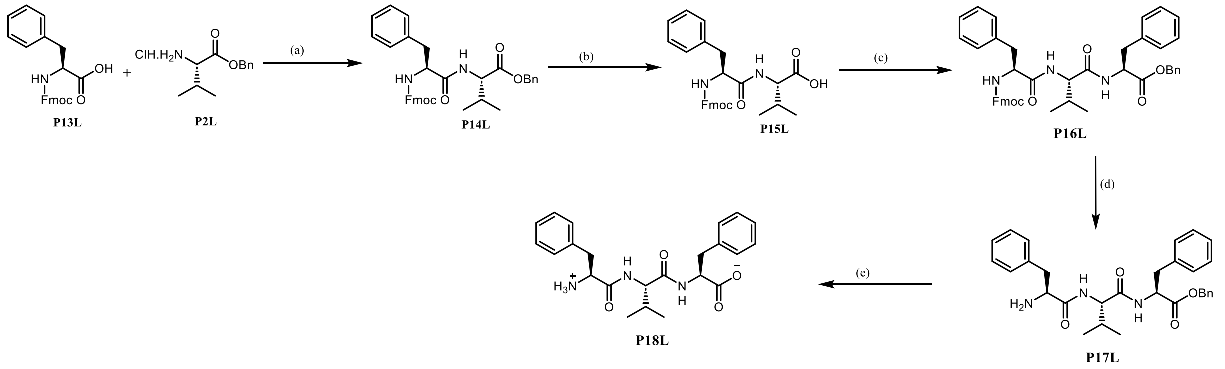

Scheme S2. Preparation of the peptide tag H_2N-L-Phe-L-Val-L-Phe-OH P18L: (a) DIPEA, HOBT, DCC, DCM, 0 °C to rt, overnight, 86%; (b) H_2/Pd(C), DCM, rt, 4h, 98%, (c) DIPEA, HOBT, HCl.NH_2-L-Phe-OBn, DCC, DCM, 0 °C to rt, overnight, 71%; (d) DBU, DCM, rt, 4h, 57%; (e) H_2/Pd(C), DCM/MeOH (1:1, v:v), rt, 3h, 52%.

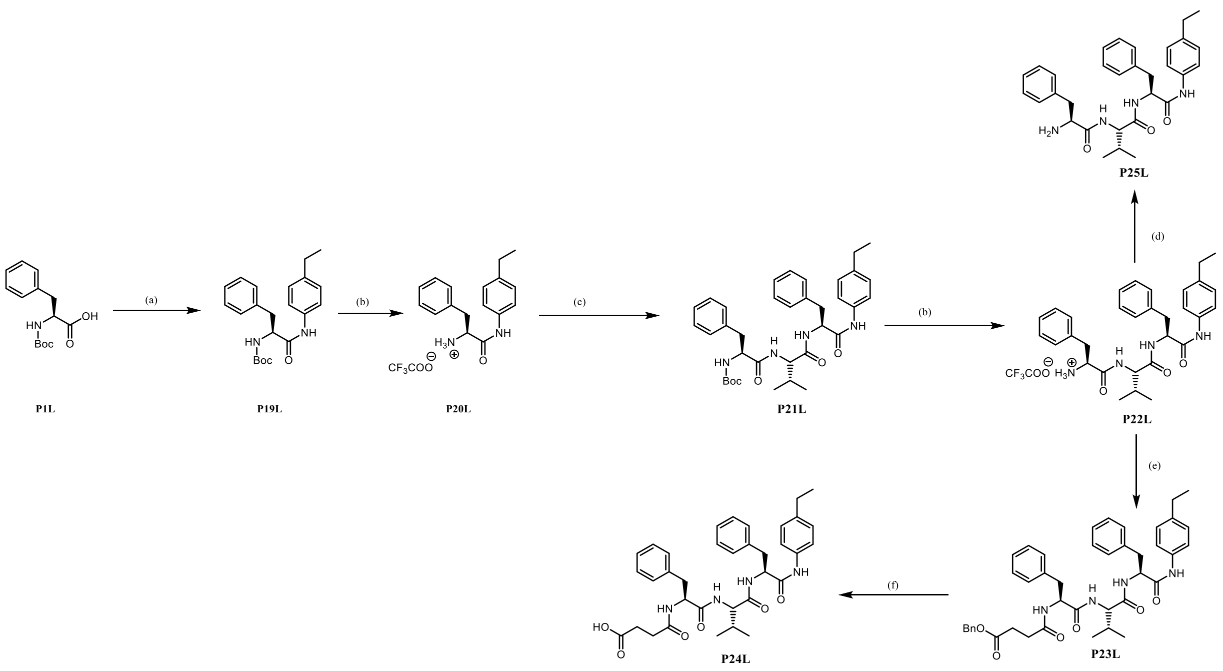

Scheme S3. Preparation of models. (a) ClCOOEt, Et_3N, 4-ethylaniline, DCM, 0 °C to -15 °C to rt, overnight, 88%; (b) CF_3COOH, DCM, rt, 1h, quantitative for P20L and 60% for P22L; (c) DIPEA, HOBT, Boc-L-Phe-L-Val-OH, DCC, DCM, 0 °C to rt, overnight, 93%; (d) NaOH 1M, DCM, 92%; (e) HOBT, HO-Suc-OBn, EDC.HCl, DCM, 0 °C to rt, overnight, 50%; (f) H_2/Pd(C), DCM/MeOH (1:1, v:v), rt, overnight, 96%.

General Salen/$MnCl_2$ alkoxyamine synthesis procedure GP2. To a stirred solution of *N,N'*-bis(salicylidene)ethylene diamine (salen ligand, .25 eq.) in THF (10 mL) is added $MnCl_2$ (0.25 eq) in an open flask. After 10 min of stirring at room temperature, a solution of Di-tert-butyl nitroxide (DBNO) (1.0 eq) and 4-vinylanilido phenylalanine-Boc (1.3 eq) in THF (50 mL) is added, and then solid $NaBH_4$ is added (4.0 eq) in four portions, every 10 minutes. The mixture is stirred between 24 h and 48 h at room temperature. at the end, the solution is dissolved in EtOAc (100 mL) in an Erlenmeyer and carefully quenched at 0 °C by addition of 1 M HCl until the solution becomes colorless or slightly orange. Then, $NaHCO_3$ is added up to neutral pH and the organic layer is washed with water (80 mL) and brine (80 mL). The organic layer is dried on $MgSO_4$, filtered, and evaporated to dryness. The purification is performed by flash column chromatography (EtOAc/ Petroleum ether).

General Boc deprotection procedure GP3: The Boc-residue (1.0 eq) is dissolved in DCM (45 mL, $V_{DCM} = 4V_{TFA}$) and TFA (10.0 eq) is added under air. The reaction mixture is stirred at room temperature. Once the substrate is fully consumed, toluene (25 mL) is added, and the solvents are removed in vacuo. The co-evaporation is repeated twice. The purification by flash column chromatography (EtOAc/ Petroleum ether) is performed.

General DCC/HOBt procedure GP4. Diisopropylethylamine (1.0 eq) is added to a stirred solution of TFA salt (1.0 eq) under argon. The mixture is stirred for 10 minutes at room temperature, then Boc-dipeptide (1.1 eq) and HOBt (1.1 eq) are added and stirred until dissolved. The mixture is cooled to 0 °C, and DCC or EDCI (1.1 eq) is added. The mixture is stirred overnight at room temperature. The mixture is filtered with cold DCM, and washed with 1 M HCl, $NaHCO_3$ (saturated solution) and brine. The purification is performed by flash column chromatography (EtOAc/ Petroleum ether).

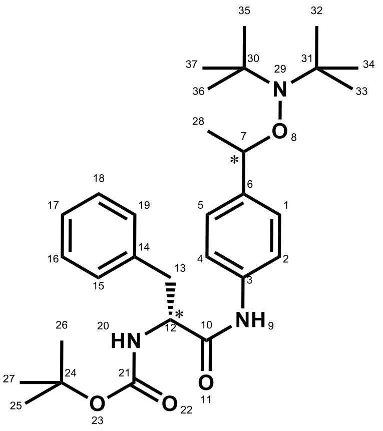

tert-butyl ((2R)-1-((4-(1-((di-tert-butylamino)oxy)ethyl)phenyl)amino)-1-oxo-3-phenylpropan-2-yl)carbamate **A9D.** By using GP2 with salen ligand (0.415 g, 1.549 mmol, 0.25 eq.), $MnCl_2$ (0.306 g, 1.549 mmol, 0.25 eq), DBNO (0.900 g, 6.196 mmol, 1.0 eq) and 4-vinylanilido phenylalanine-Boc **P3D** (2.249 g, 8.054 mmol, 1.3 eq) afforded **A9D** as a white solid (2.89 mg, 91 %, mixture of diastereomers, 1 : 1).
^{1}H NMR (300 MHz, MeOD) δ 7.44 (Broad A*B*, d, J = 8.2 Hz, 2H$_{\{2, 4\}}$), 7.31 – 7.12 (m, 7H$_{\{1, 5, 15-19\}}$), 4.77 (q, J = 6.6 Hz, 1H$_{\{7\}}$), 4.48 (m, 1H$_{\{12\}}$), 3.13 (dd, J = 13.6, 6.2 Hz, 1H$_{\{13\}}$), 2.93 (dd, J = 13.4, 8.6 Hz, 1H$_{\{13\}}$), 1.45 (d, J = 6.7 Hz, 3H$_{\{28\}}$), 1.37 (s, 9H$_{\{25-27\}}$), 1.31 (s, 9H$_{\{32-34\}}$), 1.03 (s, 9H$_{\{35-37\}}$). ^{13}C NMR (75 MHz, MeOD) δ 172.5 (C$_{\{10\}}$), 157.5 (C$_{\{21\}}$), 142.4 (C$_{\{3\}}$), 138.3 (C$_{\{14\}}$), 138.1 (C$_{\{6\}}$), 130.4 (2CH$_{\{16, 18\}}$), 129.4 (2CH$_{\{1, 5\}}$), 128.4 (2CH$_{\{15, 19\}}$), 127.9 (CH$_{\{17\}}$), 121.1 (2CH$_{\{2, 4\}}$), 83.7 (CH$_{\{7\}}$), 80.6 (C$_{\{24\}}$), 63.0 (2C$_{\{30, 31\}}$), 57.9 (CH$_{\{12\}}$), 39.8 (CH$_{2\{13\}}$), 31.8 (6CH$_{3\{32-37\}}$), 28.7 (3CH$_{3\{25-27\}}$), 22.9 (CH$_{3\{28\}}$). HRMS (ESI) : m/z calc for $C_{30}H_{46}N_3O_4^+$: 512.3483 [M+H]$^+$; found : 512.3480.

Chiral HPLC report

Column : Chiralpak IG ; mobile phase : Heptane/ethanol/dichloromethane (80/10/10), 1 mL/min

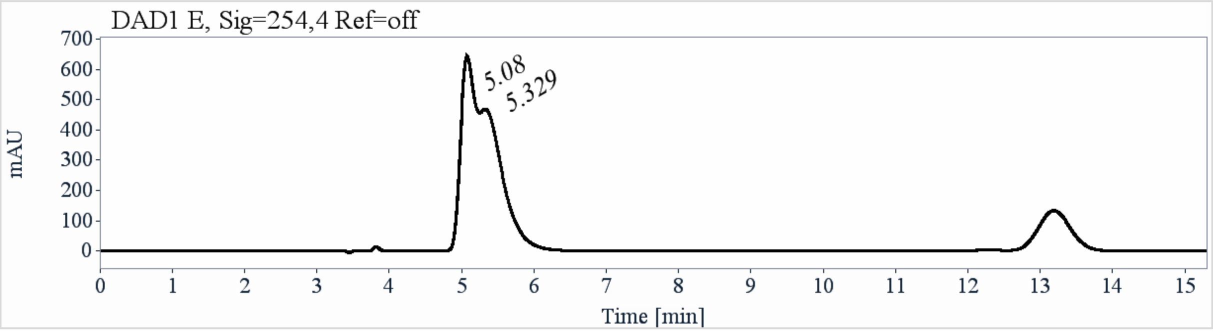

^{1}H NMR (300 MHz, MeOD)

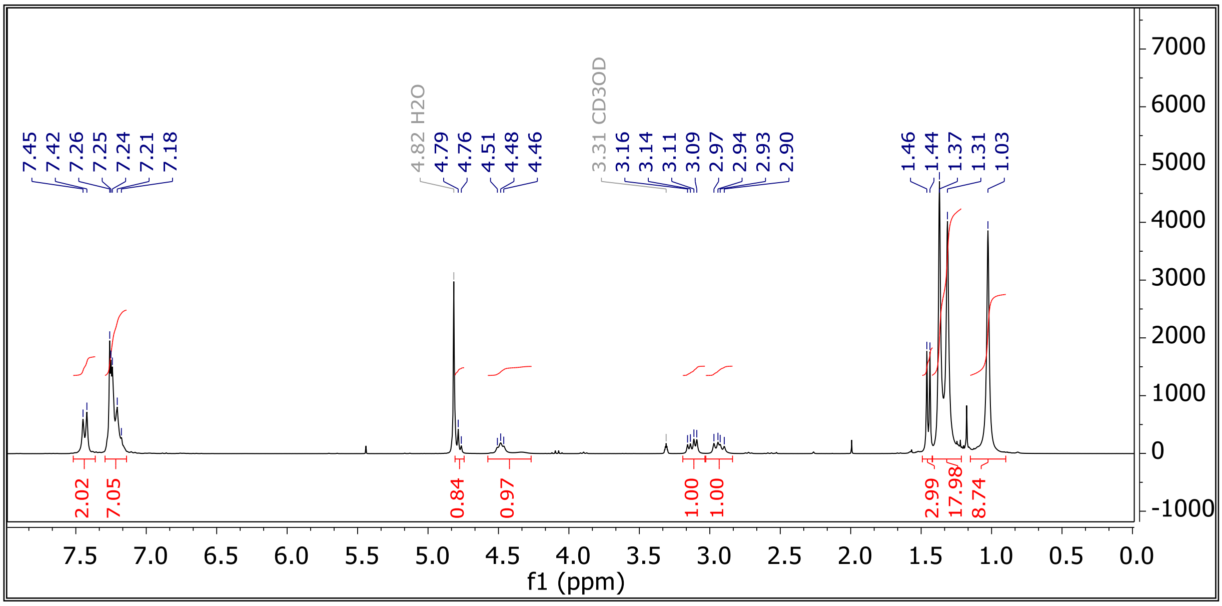

^{13}C NMR (75 MHz, MeOD)

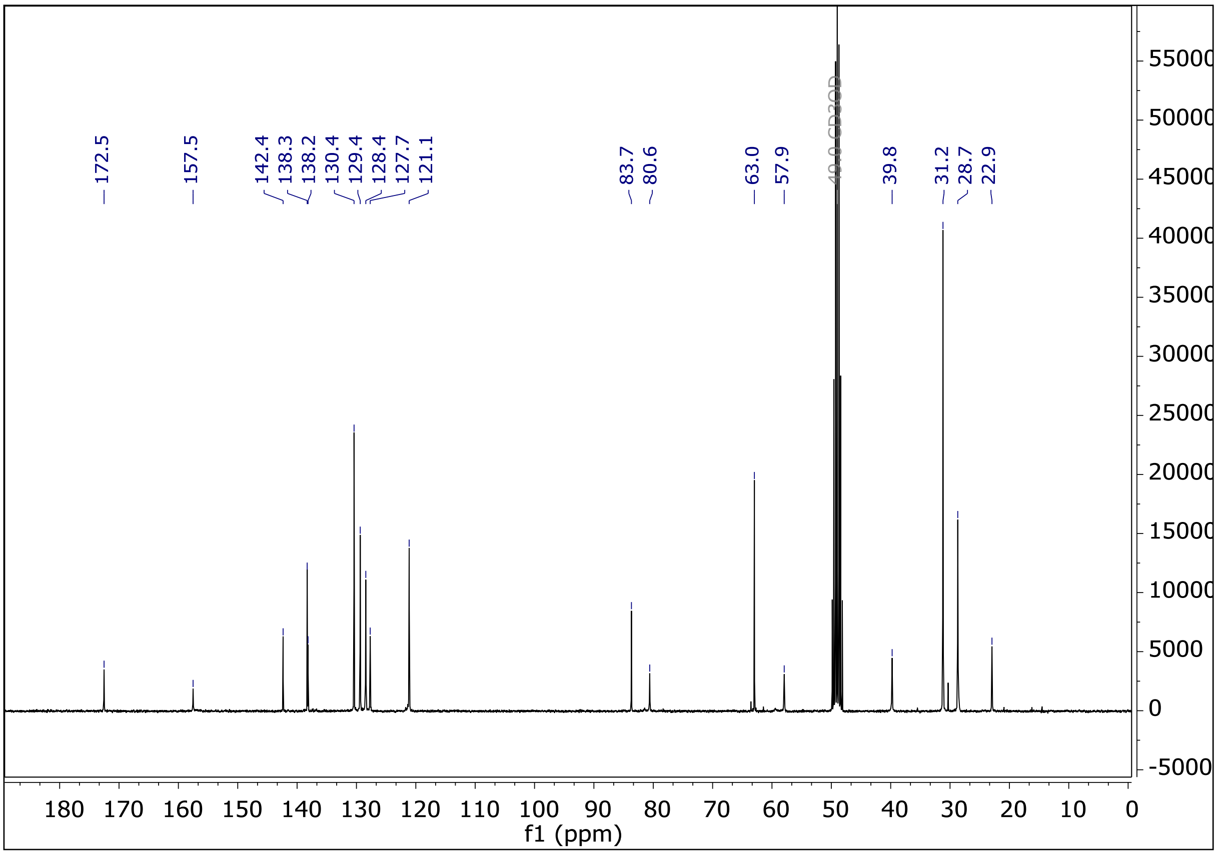

DEPT135 (75 MHz, MeOD)

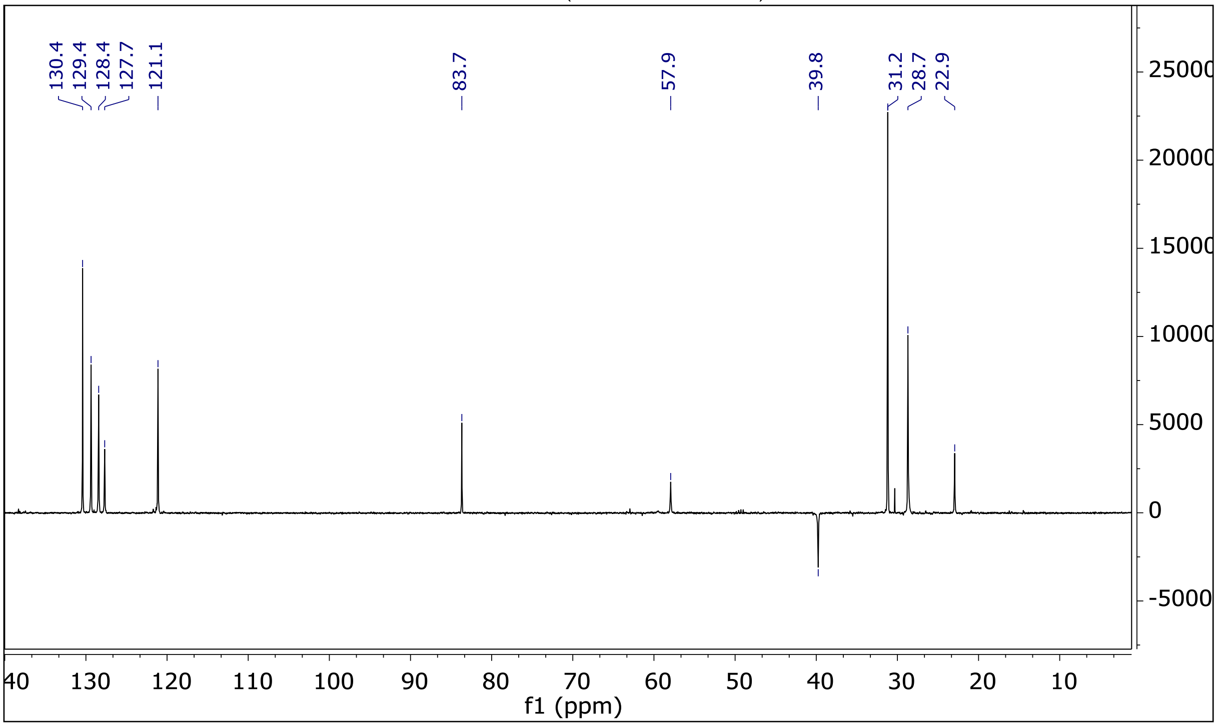

HRMS (ESI)

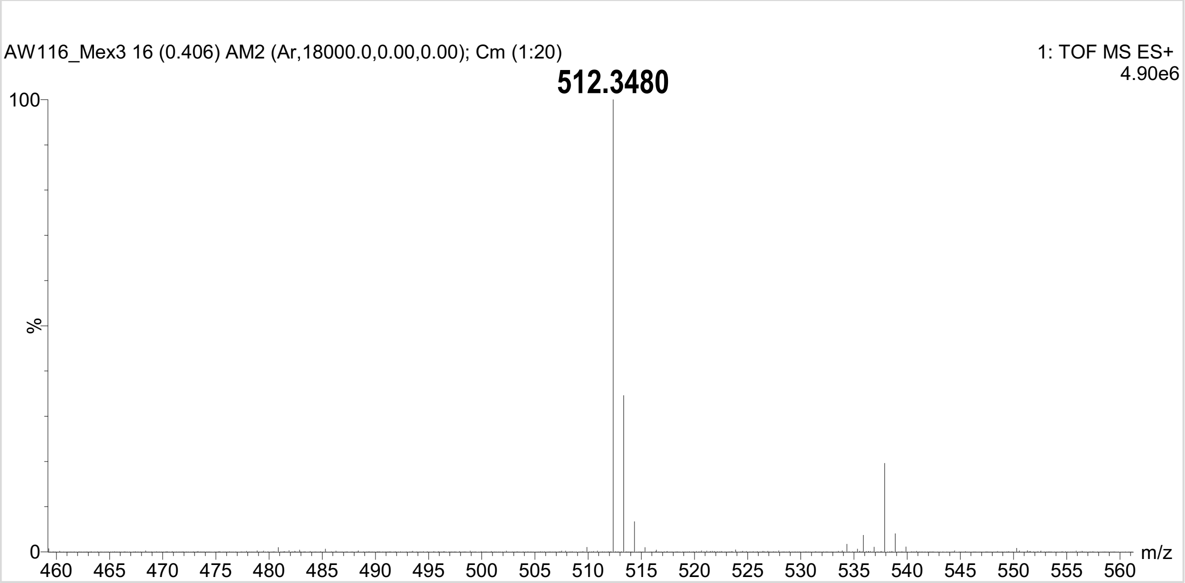

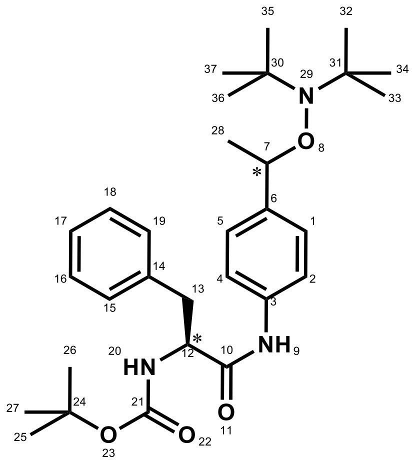

tert-butyl ((2S)-1-((4-(1-((di-tert-butylamino)oxy)ethyl)phenyl)amino)-1-oxo-3-phenylpropan-2-yl)carbamate **A9L.** By using GP2 with salen ligand (0.415 g, 1.549 mmol, 0.25 eq.), $MnCl_2$ (0.306 g, 1.549 mmol, 0.25 eq), DBNO (0.900 g, 6.196 mmol, 1.0 eq) and 4-vinylanilido phenylalanine-Boc **P3L** (2.249 g, 8.054 mmol, 1.3 eq) afforded **A9L** as a white solid (1.587 mg, 50 %, mixture of diastereomers, 1 : 1).

^{1}H NMR (300 MHz, $CDCl_3$) δ 8.86 (s, 1H$_{\{9\}}$), 7.39 (m, 2H$_{\{2, 4\}}$), 7.24 (m, 7H$_{\{1, 5, 15\text{-}19\}}$), 5.90 (d, J = 7.3 Hz, 1H$_{\{20\}}$), 5.08 – 4.55 (overlapped signals : m, 2H$_{\{12, 7\}}$), 3.15 (m, 2H$_{\{13\}}$), 1.48 (d, J = 6.5 Hz, 3H$_{\{28\}}$), 1.39 (s, 9H$_{\{25\text{-}27\}}$), 1.34 (s, 9H$_{\{32\text{-}34\}}$), 1.06 (s, 9H$_{\{35\text{-}37\}}$). ^{13}C NMR (75 MHz, $CDCl_3$) δ 170.4 (C$_{\{10\}}$), 156.1 (C$_{\{21\}}$), 141.4 (C$_{\{3\}}$), 137.0 (C$_{\{14\}}$), 136.4 (C$_{\{6\}}$), 129.4 (2CH$_{\{16, 18\}}$), 128.6 (2CH$_{\{1, 5\}}$), 127.4 4 (2CH$_{\{15, 19\}}$), 126.8 (CH$_{\{17\}}$), 119.8 (2CH$_{\{2, 4\}}$), 82.4 (CH$_{\{7\}}$), 80.2 (C$_{\{24\}}$), 61.9 (2C$_{\{30\}}$), 61.8 (C$_{\{31\}}$), 56.9 (CH$_{\{12\}}$), 38.9 (CH$_{2\{13\}}$), 30.7 (6CH$_{3\{32\text{-}37\}}$), 28.3 (3CH$_{3\{25\text{-}27\}}$), 22.6 (CH$_{3\{28\}}$). HRMS (ESI) : m/z calc for $C_{30}H_{46}N_3O_4^+$: 512.3483 [M+H]$^+$; found : 512.3482.

^{1}H NMR (300 MHz, MeOD)

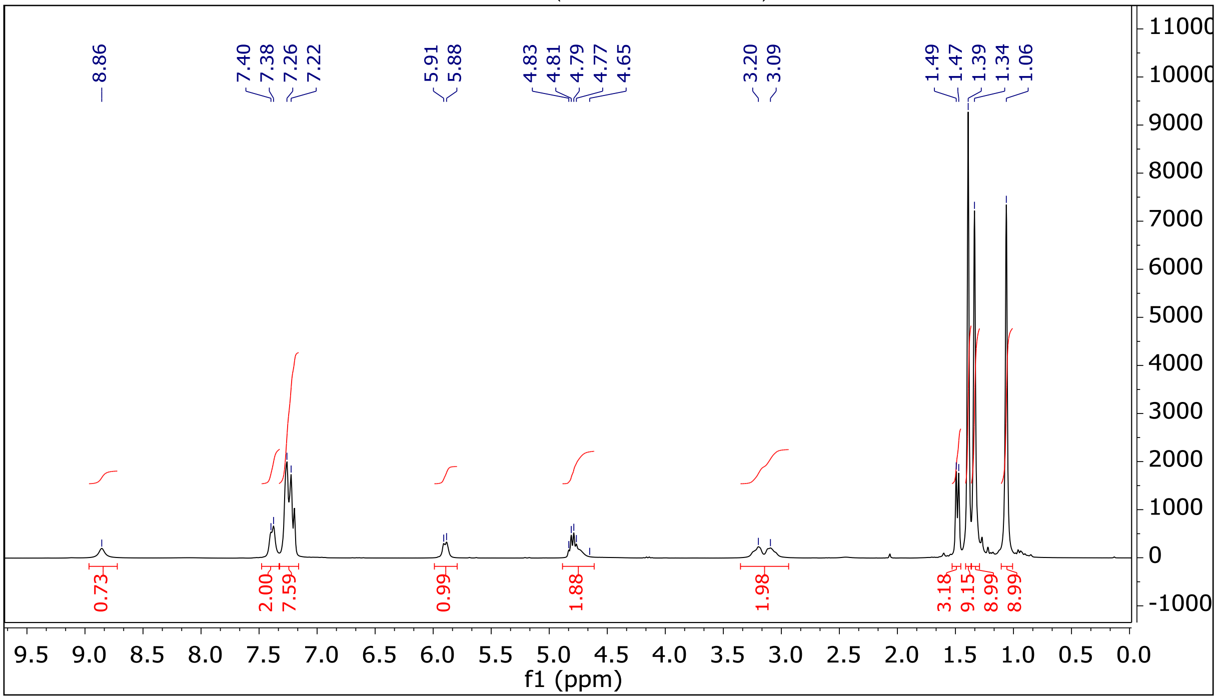

^{13}C NMR (75 MHz, $CDCl_3$)

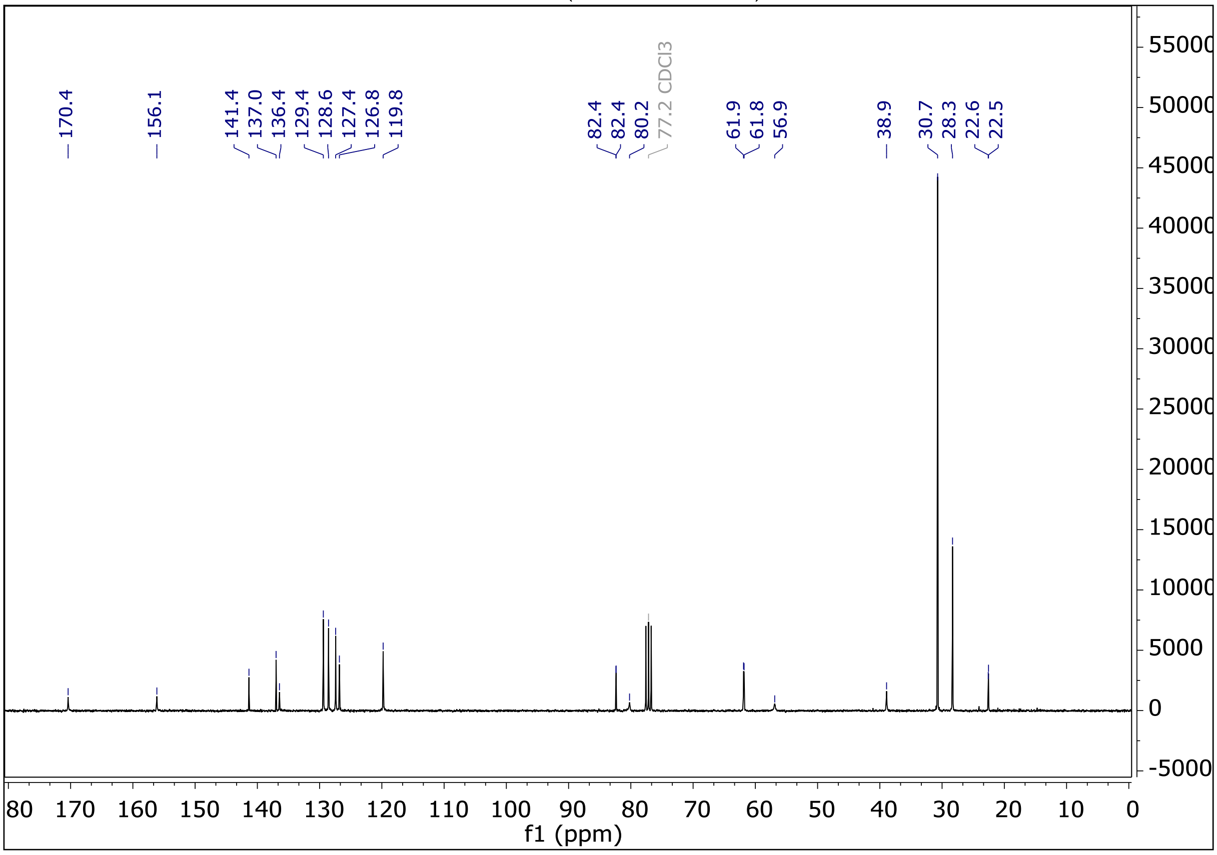

DEPT 135 (75 MHz, $CDCl_3$)

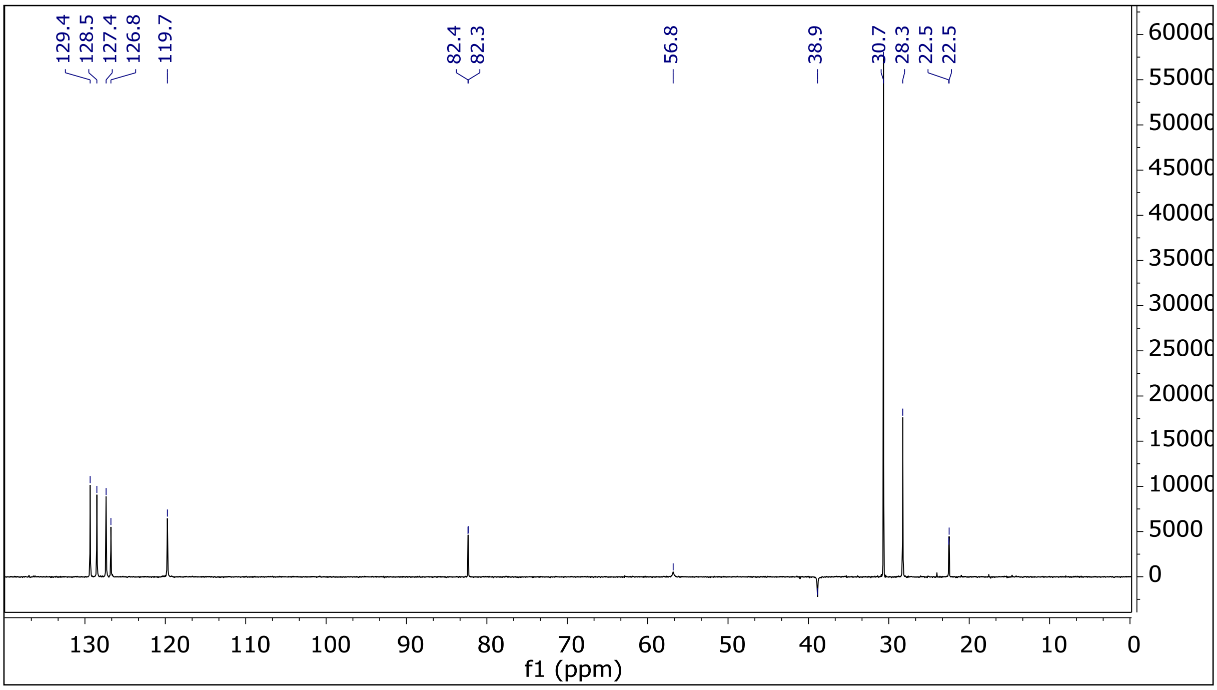

HRMS (ESI)

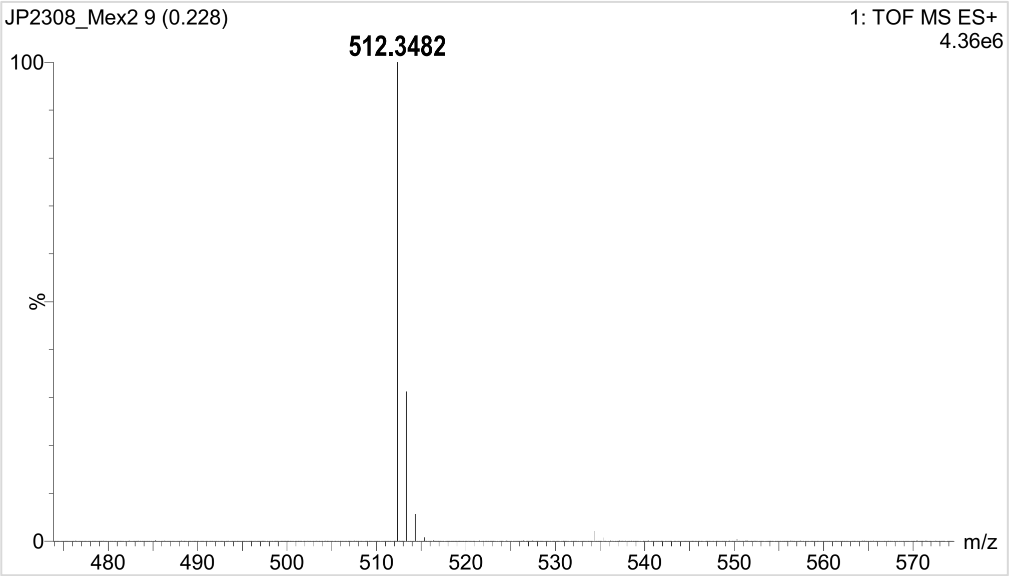

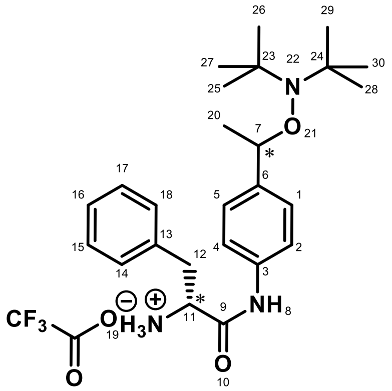

(2R)-1-((4-(1-((di-tert-butylamino)oxy)ethyl)phenyl)amino)-1-oxo-3-phenylpropan-2-aminium trifluoroacetate **A10D.** By using GP3 with **A9D** (0.484 g, 0.946 mmol, 1.0 eq) afforded **A10D** as a white solid (0.40 g, 81 %). The product was directly used in the coupling reaction without characterization.

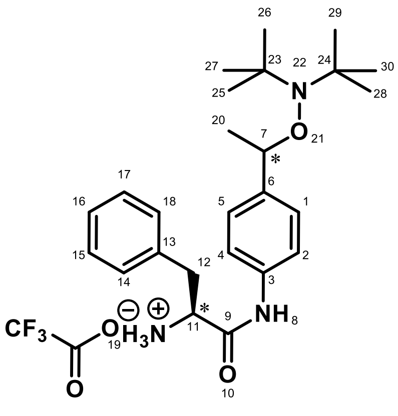

(2S)-1-((4-(1-((di-tert-butylamino)oxy)ethyl)phenyl)amino)-1-oxo-3-phenylpropan-2-aminium trifluoroacetate **A10L.** By using GP3 with **A9L** (0.484 g, 0.946 mmol, 1.0 eq) afforded **A10L** as a white solid (0.42 g, 84 %). The product was directly used in the coupling reaction without characterization.

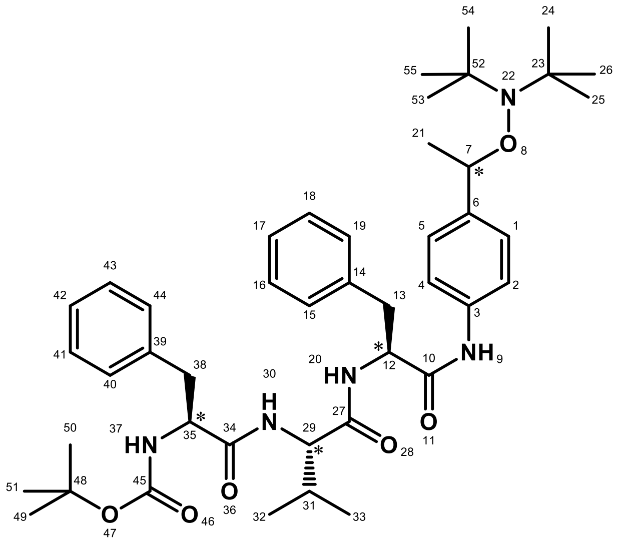

tert-butyl ((2S)-1-(((2S)-1-(((2S)-1-((4-(1-((di-tert-butylamino)oxy)ethyl)phenyl)amino)-1-oxo-3-phenylpropan-2-yl)amino)-3-methyl-1-oxobutan-2-yl)amino)-1-oxo-3-phenylpropan-2-yl)carbamate **A8L.** Using GP4 with **A10L** (1.100 g, 2.094 mmol, 1.0 eq) and DCC was replaced by EDCI.HCl to prevent the formation of DCU (a by-product that is difficult to remove entirely) affords crude **A8L**. The Purification by flash column chromatography (EtOAc/ Petroleum ether) of the crude product afforded a white solid (1.28 g, 81 %). ^{1}H NMR (400 MHz, MeOD) δ 7.38 (Broad, *A*B, d, J = 8.2 Hz, 2H$_{\{2, 4\}}$), 7.27 – 7.10 (m, 12H $_{\{1, 5, 15\text{-}19, 40\text{-}44\}}$), 4.78 (overlapped signals : water pic and q, J = 6.6 Hz, 1H$_{\{7\}}$), 4.69 (t, J = 7.6 Hz, 1H$_{\{12\}}$), 4.56 (dd, J = 10.1, 4.5 Hz, 1H$_{\{35\}}$), 4.41 (d, J = 6.7 Hz, 1H$_{\{29\}}$), 3.16 (dd, J = 13.5, 7.7 Hz, 1H$_{\{13\}}$), 3.03 (dd, J = 13.9, 4.5 Hz, 1H$_{\{38\}}$), 2.97 (dd, J = 13.7, 7.6 Hz, 1H$_{\{13\}}$), 2.78 (dd, J = 13.8, 10.0 Hz, 1H$_{\{38\}}$), 2.03 (dq, J = 6.8 Hz, 1H$_{\{31\}}$), 1.44 (d, J = 6.6 Hz, 3H$_{\{21\}}$), 1.31 (overlapped signals, s, 18H$_{\{49\text{-}51, 53\text{-}55\}}$), 1.01 (s, 9H$_{\{24\text{-}26\}}$), 0.89 – 0,81 (overlapped signals : 2d, J = 7.0 Hz, 3H$_{\{32, 33\}}$). ^{13}C NMR (101 MHz, $CDCl_3$) δ 174.4 (C$_{\{10\}}$), 173.4 (C$_{\{27\}}$), 171.5 (C$_{\{34\}}$), 157.6 (C$_{\{45\}}$), 142.4 (C$_{\{3\}}$), 138.8 (C$_{\{6\}}$), 138.0 (C$_{\{39\}}$), 137.8 (C$_{\{14\}}$), 130.4 (2CH$_{\{41, 43\}}$), 130.4 (2CH$_{\{16, 18\}}$), 129.5 (2CH$_{\{40, 44\}}$), 129.3 (2CH$_{\{15, 19\}}$), 128.3 (CH$_{\{1\}}$), 128.3 (CH$_{\{5\}}$), 127.7 (CH$_{\{42\}}$), 127.6 (CH$_{\{47\}}$), 121.3 (2CH$_{\{2,4\}}$), 83.7 (CH$_{\{7\}}$), 80.5 (C$_{\{48\}}$), 63.0 (2C$_{\{23, 52\}}$), 59.3 (CH$_{\{29\}}$), 57.1 (2CH$_{\{12, 35\}}$), 39.7 (CH$_{2\{38\}}$), 39.1 (CH$_{2\{13\}}$), 32.9 (CH$_{\{31\}}$), 31.2 (3CH$_{3\{53\text{-}55\}}$), 31.2 (3CH$_{3\{24\text{-}26\}}$), 28.8 (3CH$_{3\{49\text{-}51\}}$), 22.9 (CH$_{3\{21\}}$), 19.9 (CH$_{3\{33\}}$), 18.6 (CH$_{3\{32\}}$). HRMS (ESI) : m/z calc for $C_{44}H_{64}N_5O_6^+$: 758.4851 [M+Na]$^+$; found : 758.4856.

^{1}H NMR (400 MHz, MeOD)

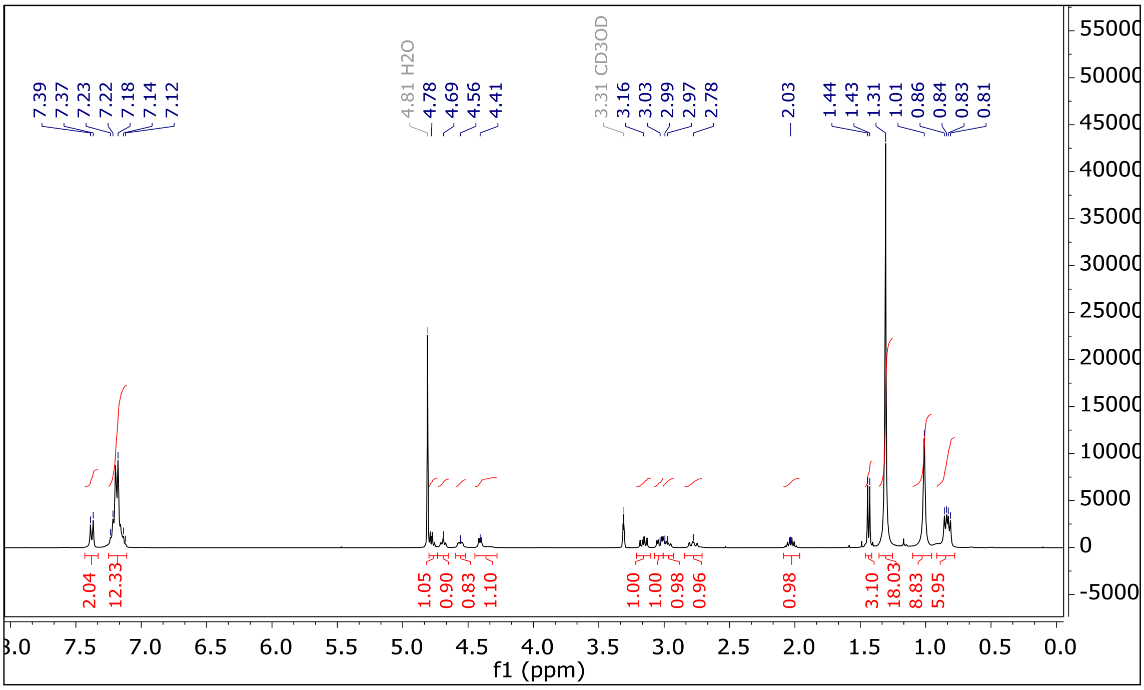

^{13}C NMR (101 MHz, MeOD)

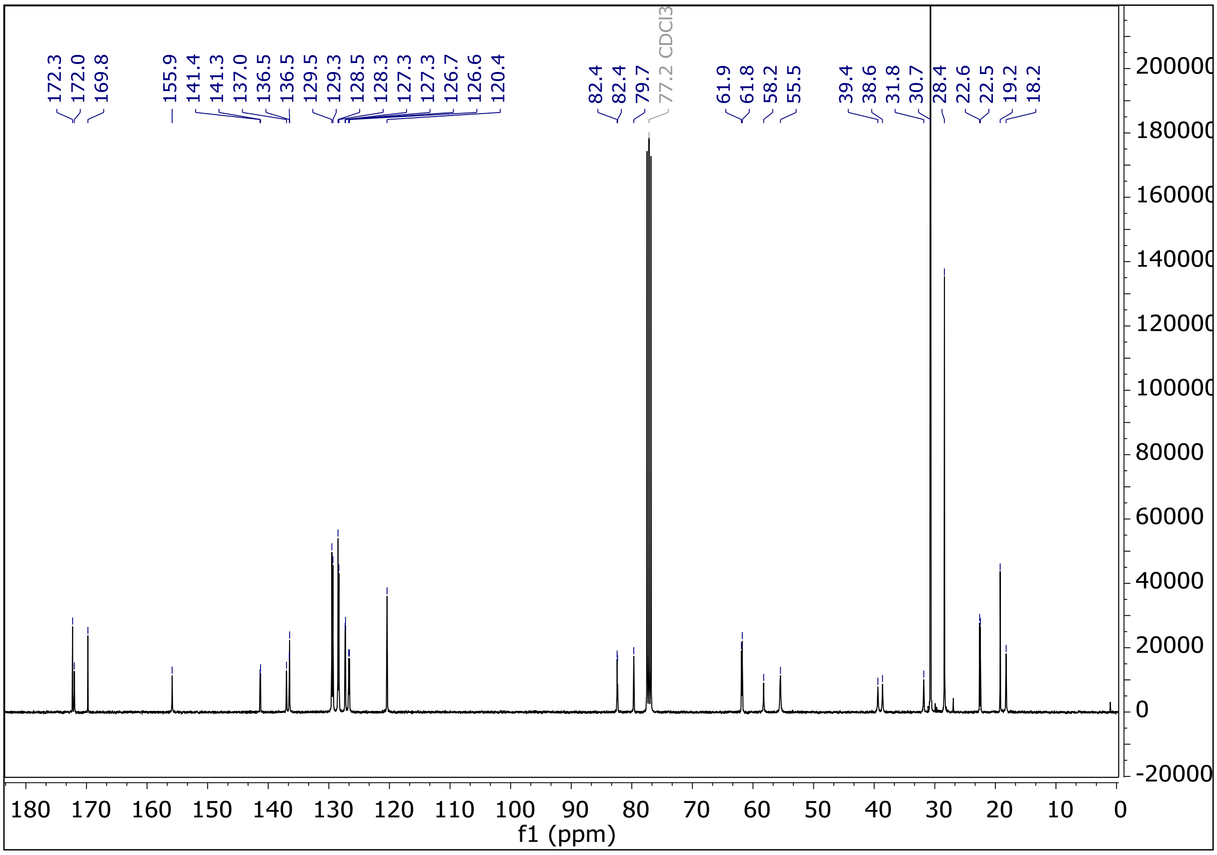

DEPT 135 (101 MHz, MeOD)

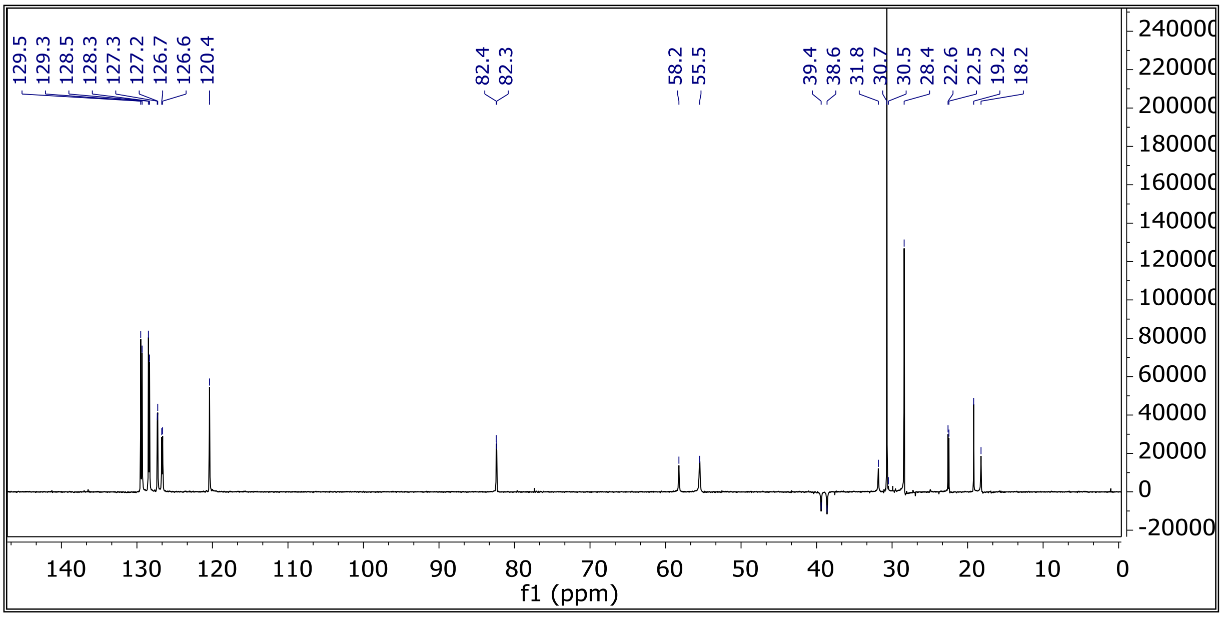

HRMS (ESI) (101 MHz, MeOD)

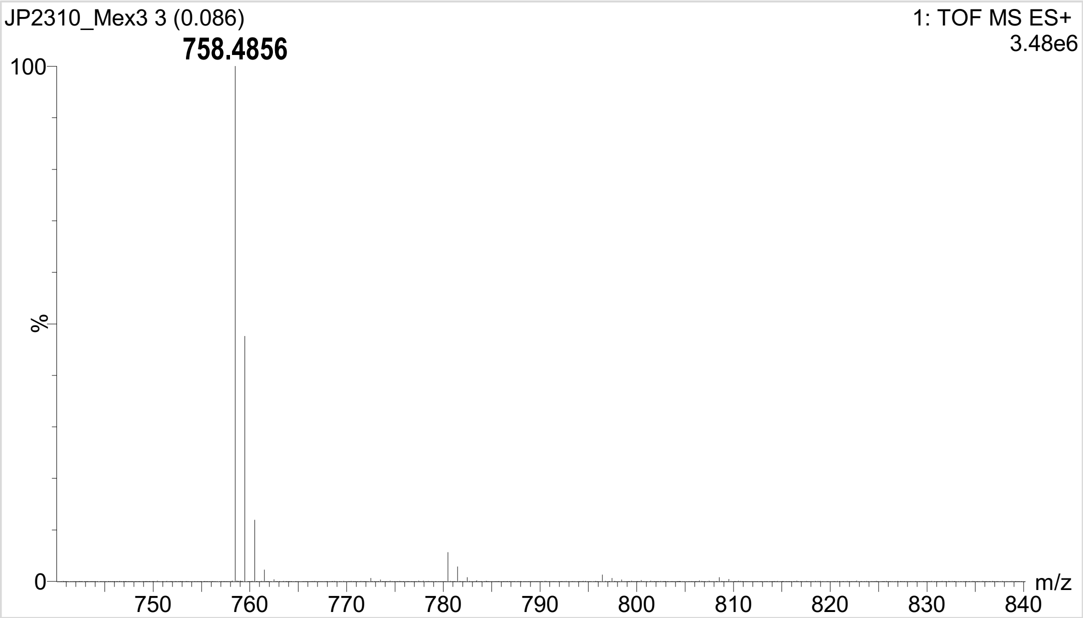

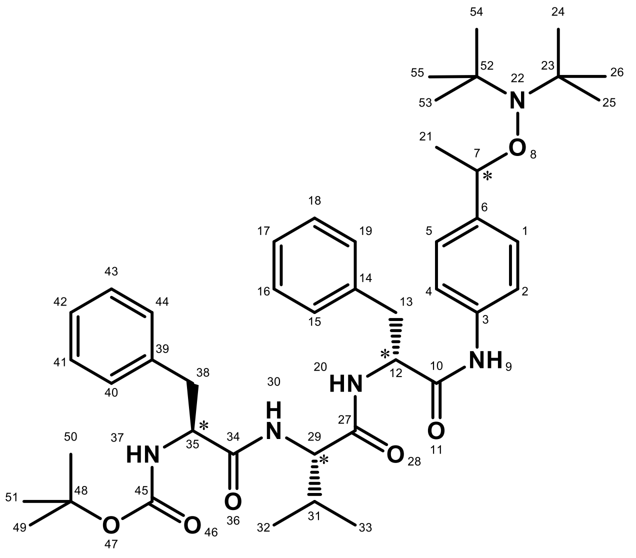

tert-butyl ((2S)-1-(((2S)-1-(((2R)-1-((4-(1-((di-tert-butylamino)oxy)ethyl)phenyl)amino)-1-oxo-3-phenylpropan-2-yl)amino)-3-methyl-1-oxobutan-2-yl)amino)-1-oxo-3-phenylpropan-2-yl)carbamate **A8DL.** Using GP4 with **A10D** (0.720 g, 1.370 mmol, 1.0 eq) and DCC was replaced by EDCI.HCl to prevent the formation of DCU (a by-product that is difficult to remove entirely) affords crude **A8DL**. The Purification by flash column chromatography (EtOAc/ Petroleum ether) of the crude product afforded a white solid (0.72 g, 69 %). ^{1}H NMR (300 MHz, MeOD) δ 7.58 (*A*B, d, J = 6.9 Hz, 2H$_{\{2, 4\}}$), 7.34 – 7.00 (m, 12H$_{\{1, 5, 15\text{-}19, 40\text{-}44\}}$), 4.88 – 4.76 (overlapped signals : q and dd, 2H$_{\{7, 35\}}$), 4.32 (dd, J = 8.5, 5.2 Hz, 1H$_{\{12\}}$), 3.99 (d, J = 7.9 Hz, 1H$_{\{29\}}$), 3.39 (dd, J = 14.0, 4.8 Hz, 1H$_{\{38\}}$), 3.06 (dd, J = 13.9, 5.1 Hz, 1H$_{\{13\}}$), 2.90 (dd, J = 14.0, 10.6 Hz, 1H$_{\{38\}}$), 2.78 (dd, J = 13.9, 8.6 Hz, 1H$_{\{13\}}$), 1.84 (dq, J = 13.6, 6.7 Hz, 1H$_{\{31\}}$), 1.46 (d, J = 6.6 Hz, 3H$_{\{21\}}$), 1.36 (s, 9H$_{\{49, 51\}}$), 1.33 (s, 9H$_{\{53, 55\}}$), 1.02 (s, 9H$_{\{24, 26\}}$), 0.80 (d, J = 6.7 Hz, 3H$_{\{33\}}$), 0.58 (d, J = 6.7 Hz, 3H$_{\{32\}}$) ^{13}C NMR (75 MHz, $CDCl_3$) δ 171.8 (C$_{\{10\}}$), 171.2 (C$_{\{27\}}$), 169.5 (C$_{\{34\}}$), 156.0 (C$_{\{45\}}$), 141.3 (C$_{\{3\}}$), 136.8 (2C$_{\{14, 39\}}$), 136.3 (C$_{\{6\}}$), 129.5 (2CH$_{\{41, 43\}}$), 129.3 (2CH$_{\{16, 18\}}$), 128.5 (2CH$_{\{40, 44\}}$), 128.2 (2CH$_{\{15, 19\}}$), 127.2 (2CH$_{\{1, 5\}}$), 126.8 (CH$_{\{42\}}$), 126.5 (CH$_{\{17\}}$), 120.4 (2CH$_{\{2, 4\}}$), 82.5 (CH$_{\{7\}}$), 79.7 (C$_{\{48\}}$), 61.9 (2C$_{\{23, 52\}}$), 58.0 (CH$_{\{29\}}$), 55.4 (CH$_{\{35\}}$), 54.9 (CH$_{\{12\}}$), 40.4 ($CH_2$$_{\{38\}}$), 40.2 ($CH_2$$_{\{13\}}$), 32.5 (CH$_{\{31\}}$), 30.7 (6$CH_3$$_{\{24\text{-}26, 53\text{-}54\}}$), 28.6 (3$CH_3$$_{\{49\text{-}51\}}$), 22.5 ($CH_3$$_{\{21\}}$), 19.2 ($CH_3$$_{\{33\}}$), 18.2 ($CH_3$$_{\{32\}}$).HRMS (ESI) : m/z calc for $C_{44}H_{64}N_5O_6^+$: 758.4851 [M+H]$^+$; found : 758.4853.

^{1}H NMR (300 MHz, MeOD

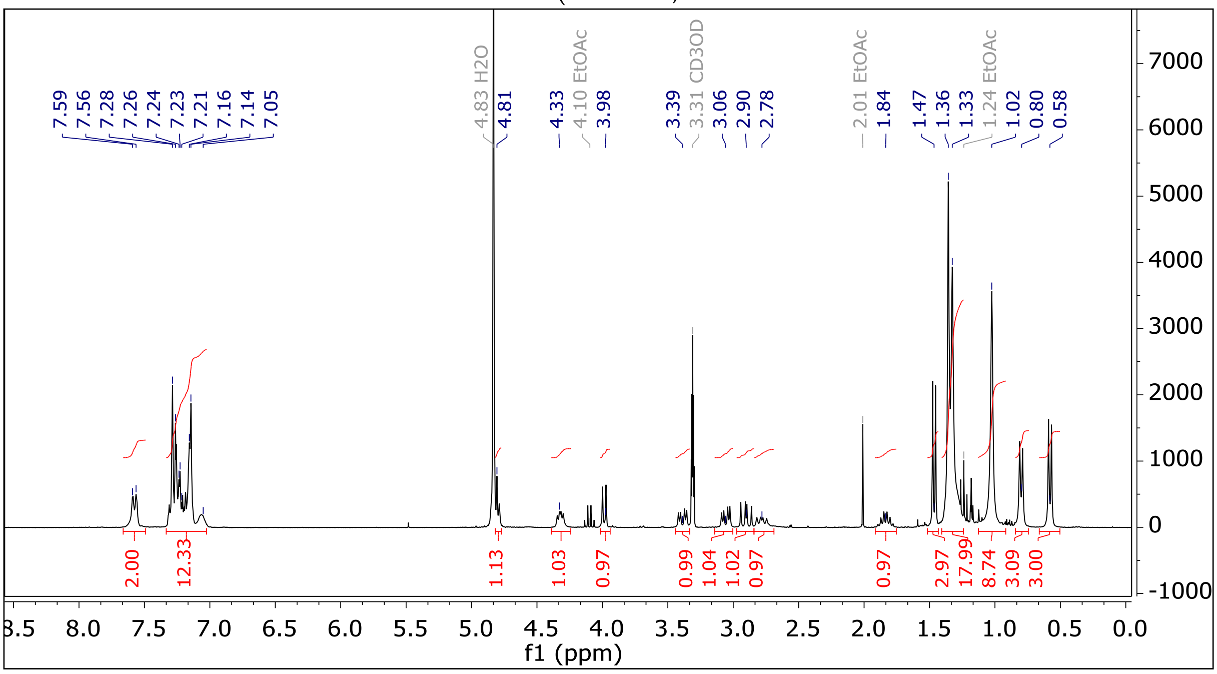

^{13}C NMR (75 MHz, $CDCl_3$)

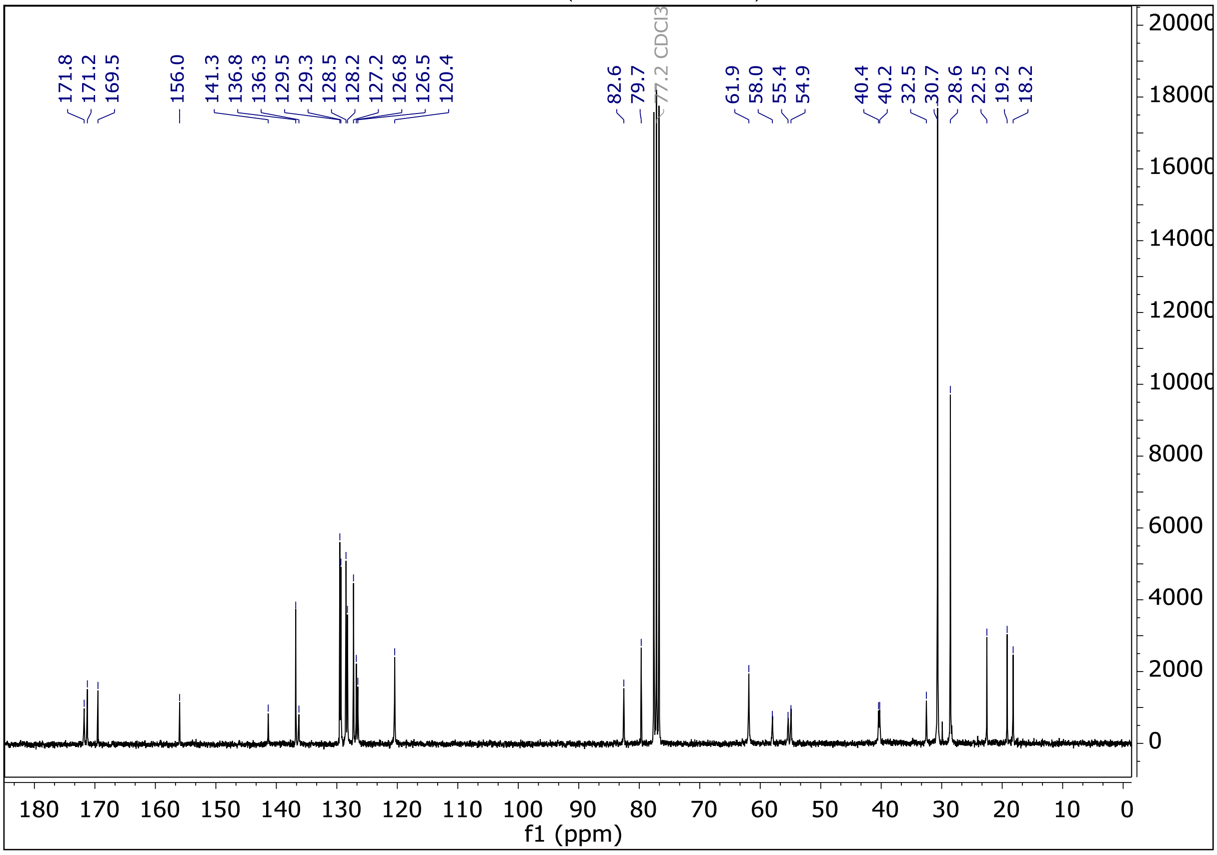

DEPT 135 (75 MHz, $CDCl_3$)

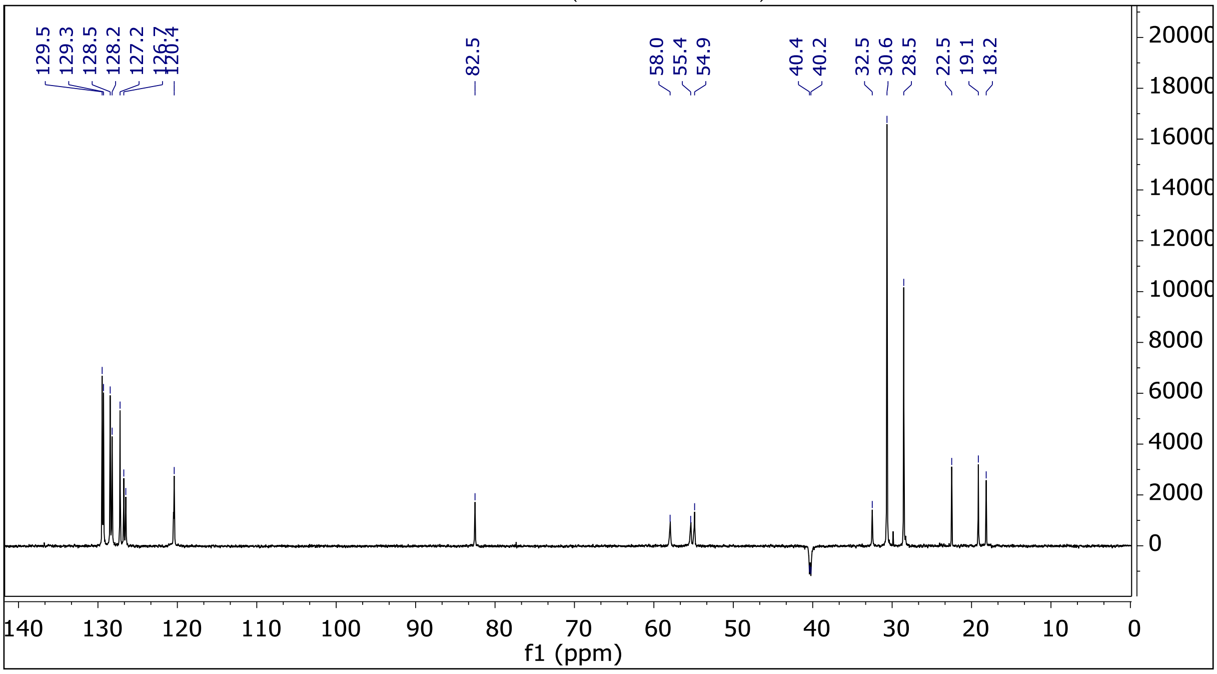

HRMS (ESI)

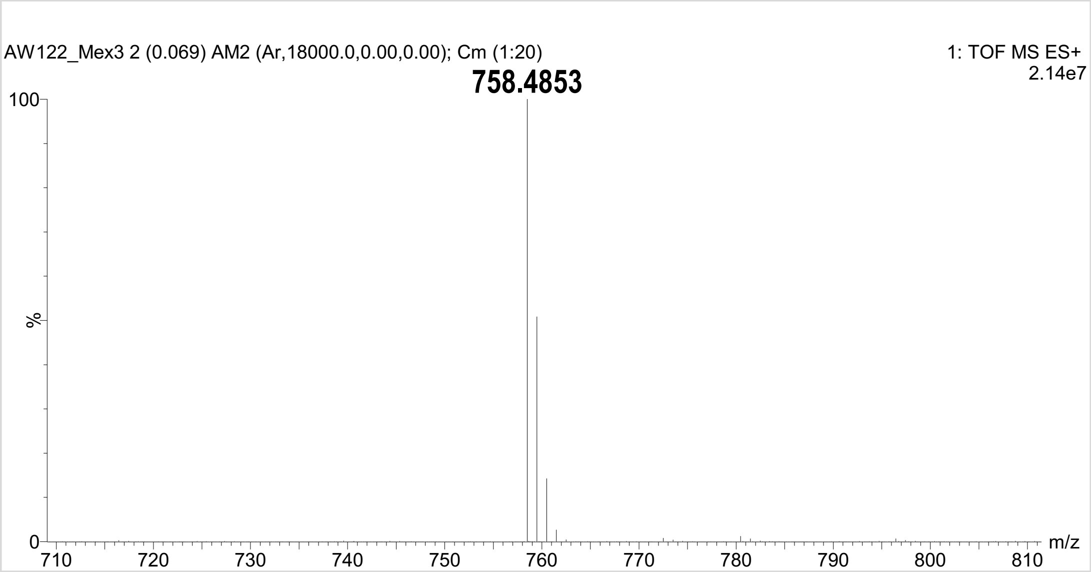
AW122_Mex3 2 (0.069) AM2 (Ar,18000.0,0.00,0.00); Cm (1:20)
1: TOF MS ES+
2.14e7
758.4853
100
%
0
710
720
730
740
750
760
770
780
790
800
810
m/z

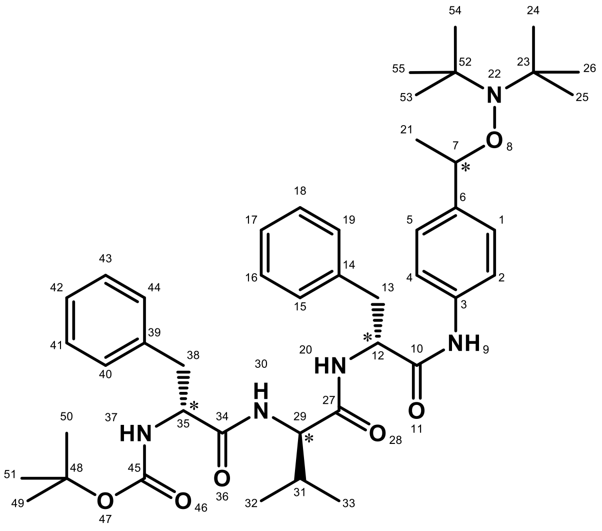

tert-butyl ((2R)-1-(((2R)-1-(((2R)-1-((4-(1-((di-tert-butylamino)oxy)ethyl)phenyl)amino)-1-oxo-3-phenylpropan-2-yl)amino)-3-methyl-1-oxobutan-2-yl)amino)-1-oxo-3-phenylpropan-2-yl)carbamate **A8D.** Using GP4 with **A10D** (1.100 g, 2.094 mmol, 1.0 eq) and DCC was replaced by EDCI.HCl to prevent the formation of DCU (a by-product that is difficult to remove entirely) affords crude **A8D**. The Purification by flash column chromatography (EtOAc/ Petroleum ether) of the crude product afforded a white solid (1.000 g, 63 %). ^{1}H NMR (400 MHz, MeOD) δ 7.41 (Broad, *A*B, d, J = 8.5 Hz, 2H$_{\{2, 4\}}$), 7.27 – 7.14 (m, 12H $_{\{1, 5, 15\text{-}19, 40\text{-}44\}}$), 4.78 (overlapped signals : water pic and q, J = 6.7 Hz, 1H$_{\{7\}}$), 4.70 (t, J = 7.5 Hz, 1H$_{\{12\}}$), 4.43 (dd, J = 9.7, 4.9 Hz, 1H$_{\{35\}}$), 4.27 (d, J = 6.9 Hz, 1H$_{\{29\}}$), 3.18 (dd, J = 13.7, 7.3 Hz, 1H$_{\{13\}}$), 3.06 (dd, J = 13.9, 4.8 Hz, 1H$_{\{38\}}$), 2.99 (dd, J = 13.6, 7.8 Hz, 1H$_{\{13\}}$), 2.79 (dd, J = 14.0, 9.7 Hz, 1H$_{\{38\}}$), 2.02 (dq, J = 6.9 Hz, 1H$_{\{31\}}$), 1.45 (d, J = 6.6 Hz, 3H$_{\{21\}}$), 1.34 (s, 9H$_{\{49\text{-}51\}}$), 1.32 (s, 9H$_{\{53\text{-}55\}}$), 1.02 (s, 9H$_{\{24\text{-}26\}}$), 0.89 – 0,81 (overlapped signals : 2d, J = 7.0 Hz, 3H$_{\{32, 33\}}$). ^{13}C NMR (101 MHz, MeOD) δ 174.4 (C$_{\{10\}}$), 173.4 (C$_{\{27\}}$), 171.5 (C$_{\{34\}}$), 157.6 (C$_{\{45\}}$), 142.4 (C$_{\{3\}}$), 138.8 (C$_{\{6\}}$), 138.0 (C$_{\{39\}}$), 137.8 (C$_{\{14\}}$), 130.4 (2CH$_{\{41, 43\}}$), 130.4 (2CH$_{\{16, 18\}}$), 129.5 (2CH$_{\{40, 44\}}$), 129.3 (2CH$_{\{15, 19\}}$), 128.3 (CH$_{\{1\}}$), 128.3 (CH$_{\{5\}}$), 127.7 (CH$_{\{42\}}$), 127.6 (CH$_{\{47\}}$), 121.3 (2CH$_{\{2,4\}}$), 83.7 (CH$_{\{7\}}$), 80.5 (C$_{\{48\}}$), 63.0 (2C$_{\{23, 52\}}$), 59.3 (CH$_{\{29\}}$), 57.1 (2CH$_{\{12, 35\}}$), 39.7 (CH$_{2\{38\}}$), 39.1 (CH$_{2\{13\}}$), 32.9 (CH$_{\{31\}}$), 31.2 (3CH$_{3\{53\text{-}55\}}$), 31.2 (3CH$_{3\{24\text{-}26\}}$), 28.8 (3CH$_{3\{49\text{-}51\}}$), 22.9 (CH$_{3\{21\}}$), 19.9 (CH$_{3\{33\}}$), 18.6 (CH$_{3\{32\}}$). HRMS (ESI) : m/z calc for $C_{44}H_{63}N_5O_6Na^+$: 780.4671 [M+Na]$^+$; found : 780.4675.

Chiral HPLC report

Column : Chiralpak IG ; mobile phase : Heptane/ethanol/dichloromethane (80/10/10), 1 mL/min

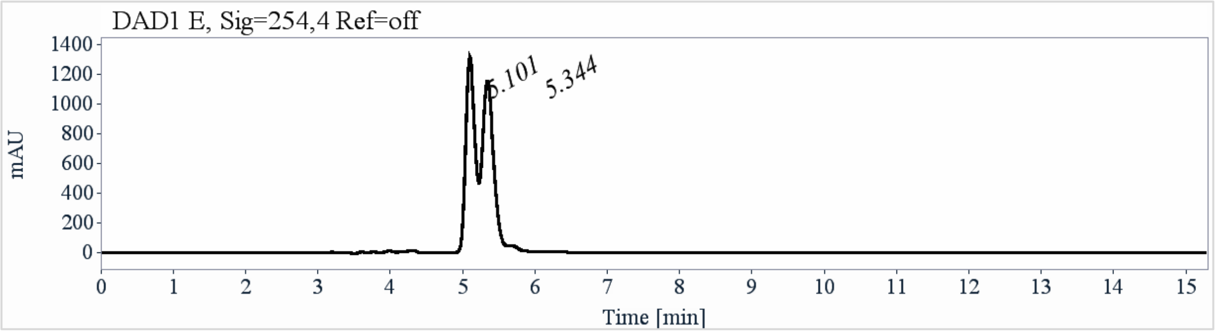

^{1}H NMR (400 MHz, MeOD)

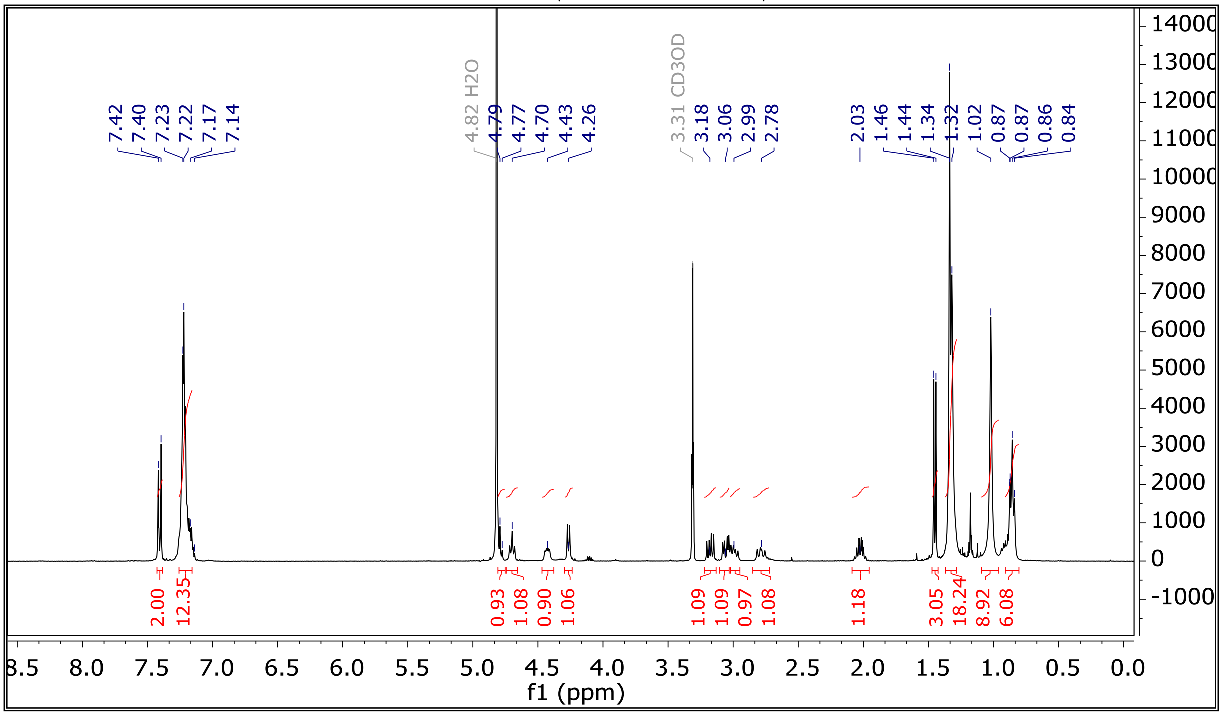

^{13}C NMR (101 MHz, MeOD)

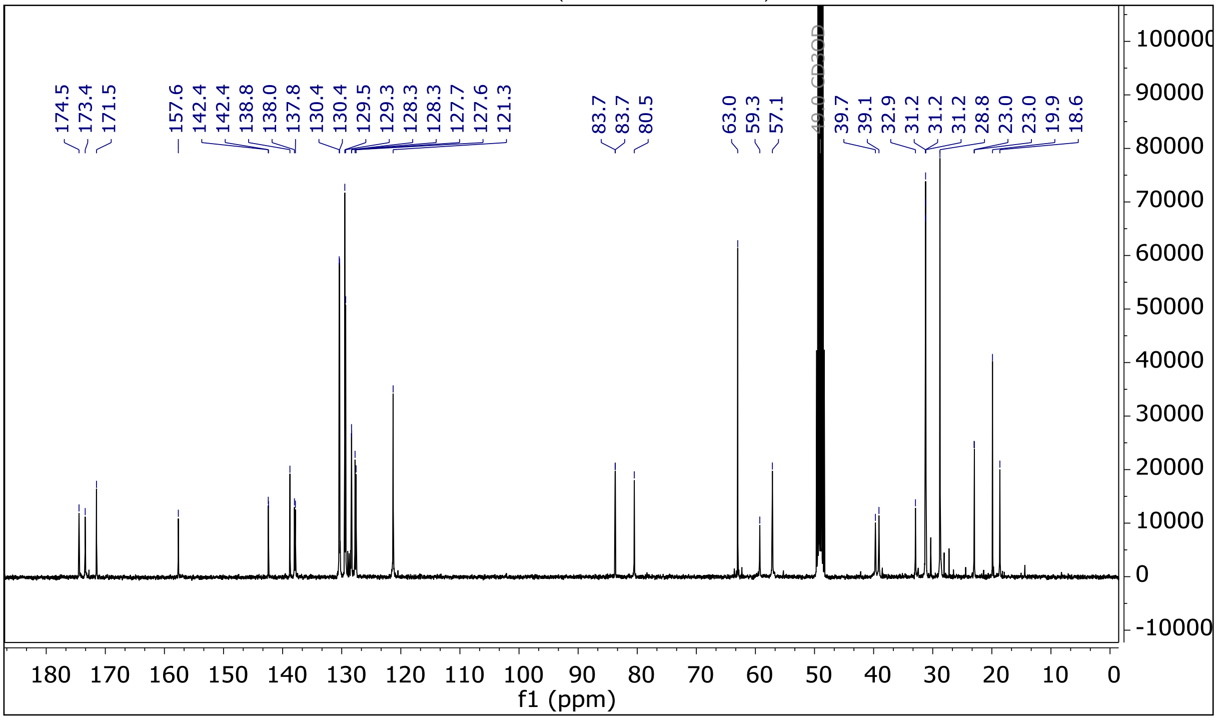

DEPT 135 (101 MHz, MeOD)

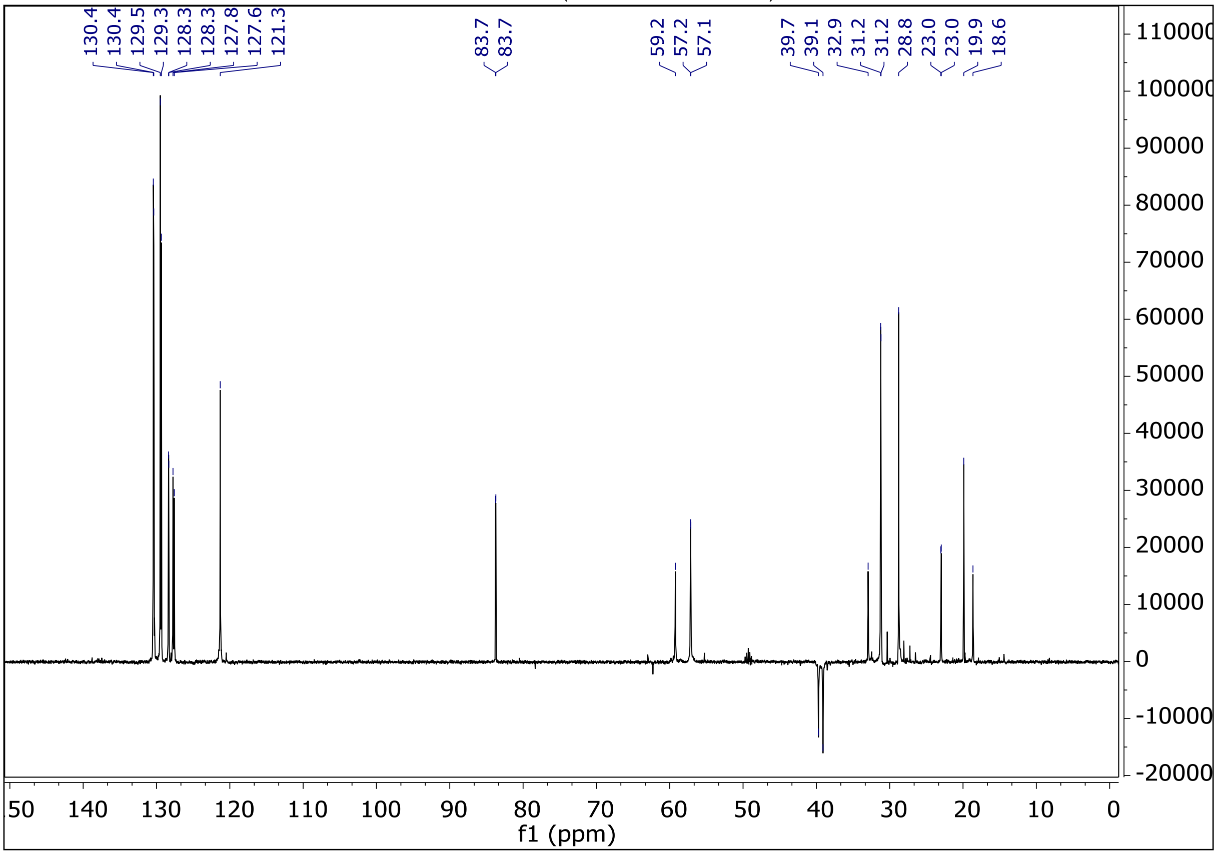

HRMS (ESI)

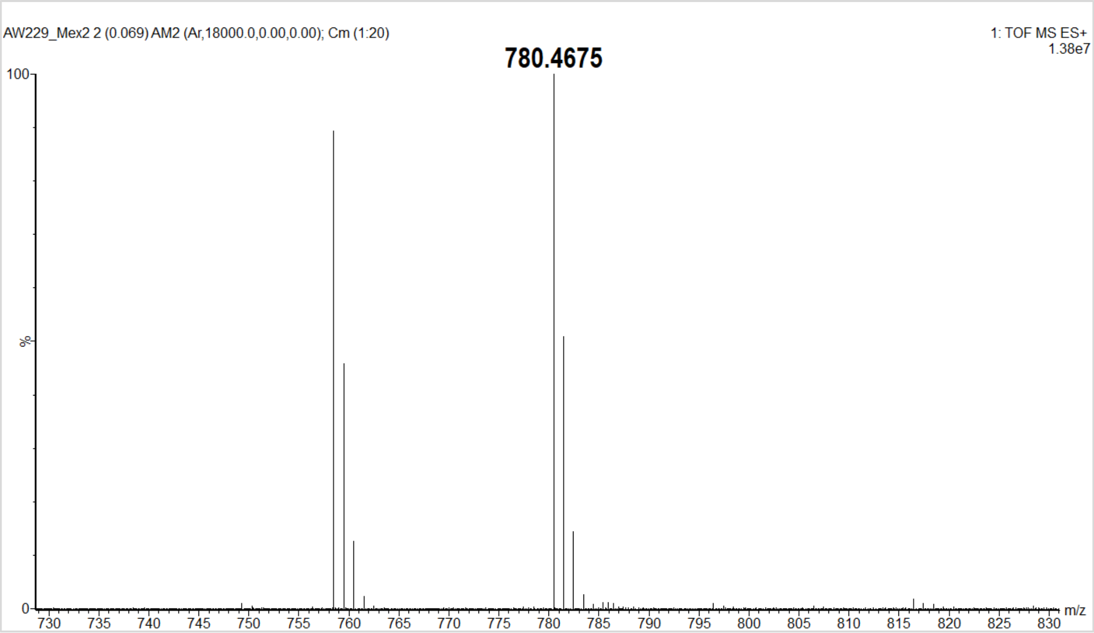

Table S1. Antimalarial activity (IC_{50}) and cytotoxicity against mammalian cells (CC_{50}) of peptide fragments.

Peptides and other comparators	Structure relationship[e]	Antiplasmodial activity[a] IC_{50} (µM)	Cytotoxicity[b] CC_{50} (µM)	Selectivity index[c] CC_{50} / IC_{50}
**P6L**	O-benzyl (Bn)<br>Boc-L-Phe-L-Val-L-Phe—O	> 50	> 50	-[d]
**P6D**	Boc-D-Phe-D-Val-D-Phe–OBn	> 50	3.2 ± 0.2	< 1
**P8L**	$CF_3COO^{\ominus}$ $H_3N^{\oplus}$—L-Phe-L-Val-L-Phe–OBn	> 50	> 50	-[d]
**P8D**	$CF_3COO^{\ominus}$ $H_3N^{\oplus}$—D-Phe-D-Val-D-Phe–OBn	> 50	> 50	-[d]
**P9L**	*N*-(Bn)succinyl-L-Phe-L-Val-L-Phe–OBn	> 50	> 50	-[d]
**P7L**	Boc-L-Phe-L-Val-L-Phe–OH	> 50	> 50	-[d]
**P7D**	Boc-D-Phe-D-Val-D-Phe–OH	> 10	> 50	-[d]

[a] IC_{50} values corresponding of 50% inhibition of *P. falciparum* growth on the strain F32-ART [26] were determined by Sybr Green assay [27]. Values correspond to the mean ± SEM of 3 to 6 independent experiments, with technical triplicate repeats carried out for each one.
[b] Cytotoxicity performed on Vero cells (cell line from kidney of a normal adult African green monkey). Values correspond to the mean ± SEM of 3 to 7 independent experiments, with technical duplicate repeats carried out for each one.
[c] Selectivity index corresponding to the cytotoxicity/antiplasmodial activity ratio.
[d] Cannot be determined on the basis of the present data.
[e] Enantiomer only refer to the configuration of the tripeptide Phe-Val-Phe.
